# 1,3-Dipolar Cycloaddition and Mannich Reactions of Alkynyl Triterpenes: New Trends in Synthetic Strategies and Pharmacological Applications

**DOI:** 10.3390/ijms26094329

**Published:** 2025-05-02

**Authors:** Anastasiya V. Petrova, Oxana B. Kazakova

**Affiliations:** Ufa Institute of Chemistry, Ufa Federal Research Centre, Russian Academy of Science, 69, Prospect Octyabrya, Ufa 450054, Russia; obf@anrb.ru

**Keywords:** natural compounds, triterpenoids, 1,2,3-triazoles, CuAAC, Mannich bases, biological activity

## Abstract

Nitrogen-containing substitutes, such as 1,2,3-triazoles and Mannich bases, are major pharmacophore systems, among others. The presented review summarizes the recent advances (2019–2024) in the synthesis of 1,2,3-triazoles and Mannich bases conjugated with a triterpenic core. These structural modifications have proven to be effective strategies for modulating the biological activity of triterpenes, with particular emphasis on antitumor and antiviral properties. Recent efforts in expanding the structural diversity of triazoles through A-ring modifications and C28 (or C30) substitutions are discussed. Notably, the first examples of N-alkylation of indole triterpenoids by propargyl bromide are presented, along with the application of propargylamine in the synthesis of rare triterpenic aldimines. The review also covers an application of triterpene alkynes in Mannich base synthesis, focusing on functionalization at various positions, including C28 and C19 of the lupane platform, and incorporating of amino acid spacers. While significant progress has been made both in synthetic strategies and pharmacological applications, further research is needed to fully explore the antibacterial, anti-inflammatory, and antidiabetic potential. The review will be useful to researchers in the fields of organic synthesis, natural product and medicinal chemistry, and pharmacology.

## 1. Introduction

The chemistry of alkyne-containing natural compounds is one of the most important and well-studied branches of medicinal chemistry. The triple bond plays an important role as part of the pharmacophore and could be used to obtain new nitrogen-containing compounds, such as 1,2,3-triazols or Mannich bases. These nitrogen-containing scaffolds offer advantages such as directly affecting the reactivity of the target skeleton, activity (or toxicology) of the compounds, interactions between target drugs and different target inhibitors, as well as being able to influence metabolism and pharmacokinetics.

Click chemistry, in particularly Cu (I)-catalyzed azide-alkyne cycloaddition (CuAAC), is a way of building compounds by linking two molecules into a 1,2,3-triazole ring. Due to its structure, which includes polarity, rigidity, and the ability to form hydrogen bonds, 1,2,3-triazole serves as an effective bioisostere, mimicking other functional groups in the synthesis of new biologically active molecules, while demonstrating increased resistance to various chemical influences [1,2]. The CuACC method has revolutionized chemical synthesis to such an extent that the Nobel Prize in Chemistry in 2022 was awarded to Barry Sharpless, Morten Meldal, and Carolyn Bertozzi for their contributions to the field.

The Mannich reaction converts organic substrates into the corresponding aminomethyl derivatives. These substrates turn into water-soluble compounds with improved bioavailability and novel interesting biological activities that have applications in many clinical conditions. The structural features and characteristics of Mannich bases allow the release of the active substance by deaminomethylation or deamination; therefore, this class is often considered in the concept of “pro-drugs” [3,4,5].

The tendency to obtain “hybrid structures” also affects a class of natural compounds, such as triterpenoids, and has led to the discovery of various molecules possessing two independent bioactive parts [6]. Research on the synthesis of new 1,2,3-triazoles of triterpenoids and steroids has been ongoing for more than 20 years, and during this time the number of publications has reached more than 100. Their use in such reactions is due to their complex polycyclic structure, which provides many positions for functionalization, as well as the fact that triterpenes often have low toxicity, which makes them promising for drug development. The presence of functional groups such as hydroxyls and carboxyls in the native molecule facilitates modifications, allowing the effective introduction of triazole or aminoalkyl substituents through ether, amide, and other bonds. All of these factors together make triterpenes ideal platforms for the creation of new biologically active compounds with improved properties. Professors Milan Urban and Rene Csuk already summed up the results of 10 years in their reviews in 2018 and 2019, which were dedicated to click reactions in the chemistry of triterpenes [7,8]. As reported in these reviews, triazoles have generally emerged as antitumor and antiviral agents. After 2019, publications included other types of activities, for example, antidiabetic, antimicrobial, antiparasitic, anti-inflammatory, and even neuroprotective effects. Making a search of the references, we analyzed the sources from the databases of Google Scholar, PubMed, and Scopus from 2019 to 2024. Thus, this review summarizes recent advances in synthetic approaches to triterpene alkynes, azides, 1,2,3-triazoles, and Mannich bases and in studies of their biological activity.

## 2. Synthesis of Triterpene Alkynes and Azides as the Key Precursors for 1,2,3-Triazoles and Mannich Bases

**Scheme 1:**
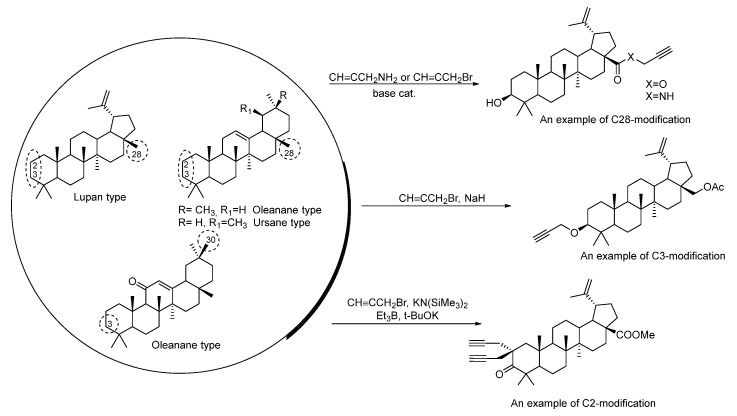
The main methods to introduce an alkyne group to the triterpene core.

The most common synthesis of triazoles is based on the conjugation of triterpene alkynes with various organic azides. Scheme 1 shows the key positions for introducing an acetylene-containing group as a substituent in the side chain. Thus, functionalization is carried out mainly at the C3 and C28 positions of the backbone, usually by reactions of hydroxy/carbonyl/carboxyl groups with propargylamine or propargyl bromide under base catalysis. α-Alkylation of 3-oxotriterpenoids with propargyl bromide in the presence of strong bases (KN(SiMe_3_)_2_, Et_3_B, and t-BuOK) forms the basis for chemoselective methods for the synthesis of C2-substituted representatives.

**Figure 1:**
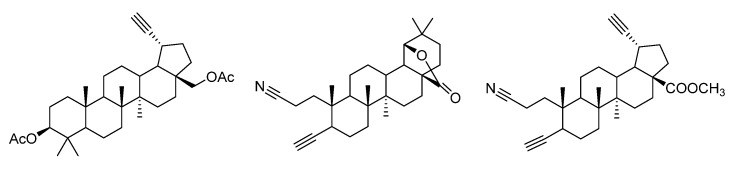
Triterpenoids with an alkyne group formed by a skeleton rearrangement.

One of the known methods to obtain acetylene derivatives includes the action of phosphorus halides on the aldehyde or ketone, followed by dehydrohalogenation of the resulting gem-dihalogen derivative. For example, 3,28-diacetoxy-29-norlupan-20-one, synthesized by ozonolysis of diacetoxybetulin, is treated with POCl_3_ to afford the C19 alkynyl triterpenoid [9], and later this reaction was extended to other triterpene methyl ketones (Figure 1).

**Figure 2:**
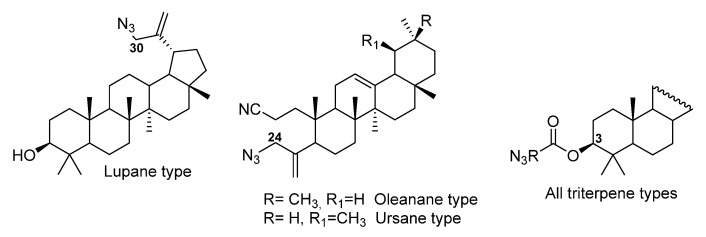
The key positions of the azide function in all triterpene-type scaffolds.

On the other hand, the synthesis of triazoles can be carried out through the triterpene-azide and alkyne pair. One of the methods to introduce an azido function is based on acylation of the hydroxyl group with azido acids. The second option includes allylic bromination of the isopropenyl group, followed by treatment with sodium azide. But this method has gained popularity only for lupanes, which possess a methyl group in the alpha-position to the exo-double bond. For oleananes and ursanes, the method of adding an azide function involves the opening of the A-ring by the Beckmann II reaction (Figure 2).

**Figure 3:**
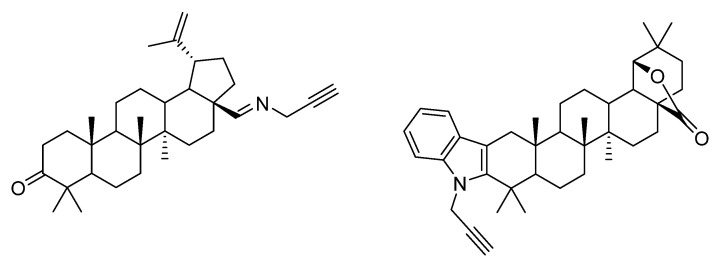
Rare examples of *N*-substituted triterpenic alkynes.

Although nucleophilic substitution reactions at the carbonyl group have been used previously, the introduction of an imine-alkyne group was described recently [10]. In 2019, propargylation of the indole ring connected with a lupane triterpene core was first described [11] and then developed with other types of triterpenes [12] (Figure 3).

All of the described approaches have been applied to the synthesis of 1,2,3-triazoles and Mannich base derivatives.

## 3. Modification of Alkynyl-Triterpenoids by the Click Reaction

**Figure 4:**
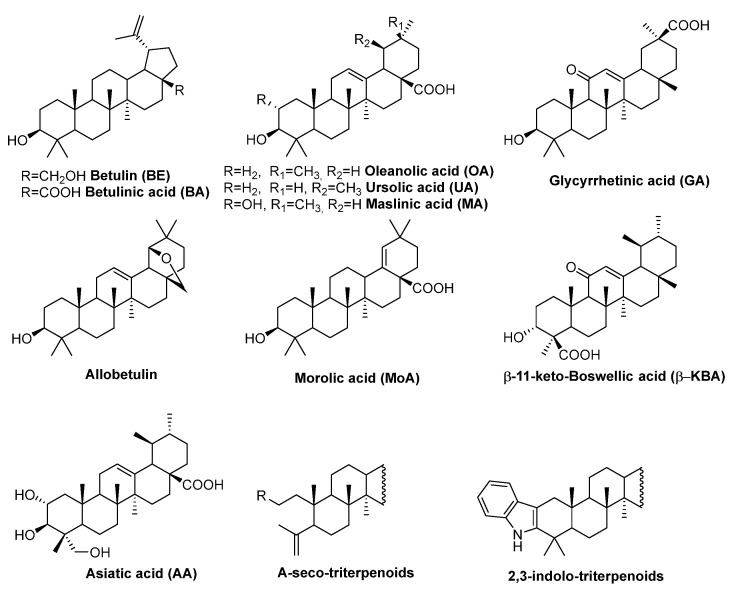
The main starting triterpene scaffolds for 1,2,3-triazole synthesis.

Modification of alkynyl and azido triterpenoids by the click reaction method is one of the popular methods for obtaining biologically active molecules. Previously, the hybrid structures of triterpenes and a bioactive subunit linked by a triazole ring were already reviewed [7,8]. As expected, these molecules were, in most cases, more active than the parent compounds, but not as much as aimed. Over the past 5 years, research on conjugates of triterpenoids with triazoles has continued. In some cases, the authors have managed to advance the research; for example, some work contained in vivo data, which was not reported before. This review will consider modifications of triterpenoids of lupanes, oleananes, ursane types, and some A-ring modified analogs. The structures of the main starting platforms are outlined in Figure 4.

### 3.1. Modifications at C28/C30 Carboxylic Position

Despite the developments in triterpene chemistry using functionalization at all available positions, the carboxyl group (C28/C30 mainly) still remains the most used functional group for modification. These are mainly derivatives with an ester group and less often an amide group.

**Figure 5:**
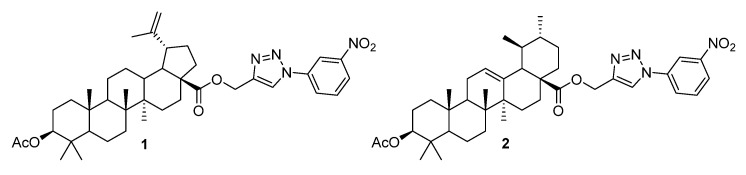
Betulinic and ursolic acid C28 ester 1,2,3-triazoles.

Based on acetoxy-betulinic or acetoxy-ursolic acid propargyl esters, triazoles **1–2** with a nitroaryl group were synthesized as new drugs against respiratory syncytial virus (RSV) (Figure 5) [13]. Compound **2** was the most effective, with an EC_50_ value of 0.053 μM, TI of 11,160.37, and it inhibited hRSV F protein gene expression by approximately 65%.

**Figure 6:**
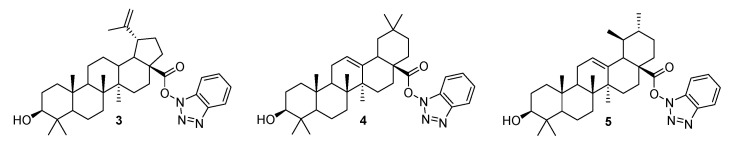
Betulinic, oleanolic, and ursolic acid C28 benzotriazole esters.

The synthesis and biological assessment of benzotriazole esters of BA, OA, and UA 3-5 were reported [14]. Compounds **3–5** were obtained using DCC-coupled esterification reactions (Figure 6). The all-tested compounds demonstrated dose-dependent toxicity against melanoma cells while sparing healthy keratinocytes, inducing characteristic apoptotic changes like nuclear fragmentation and membrane disruption. This pro-apoptotic effect was confirmed by decreased expression of anti-apoptotic Bcl-2 and increased expression of pro-apoptotic Bax. Furthermore, these compounds inhibited mitochondrial function, and molecular docking indicated enhanced binding affinity to Bcl-2 compared to related triterpenoids, resembling known Bcl-2 inhibitors.

**Figure 7:**
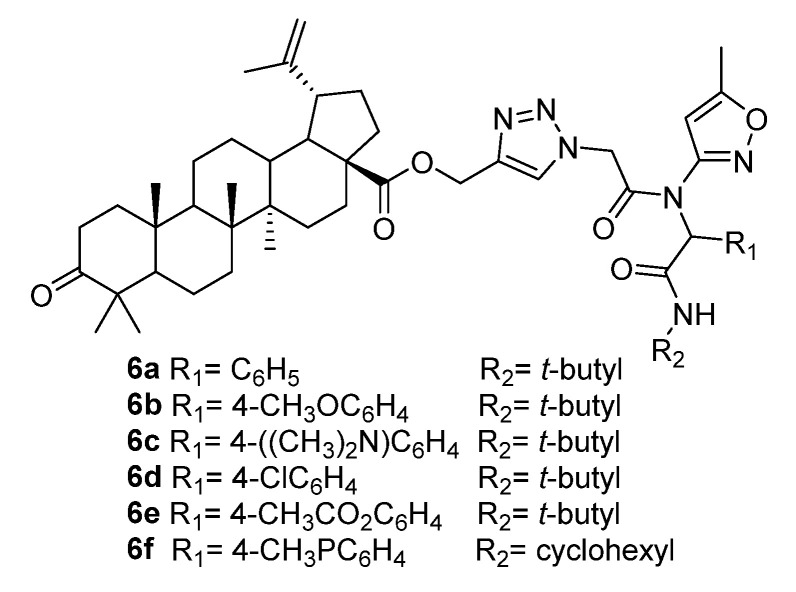
Betulonic acid hybrids with heterocycle peptidomimetic fragments at C28.

A synthetic route to hybrid molecules of **3-oxo-BA** propargyl ester linked with a peptidomimetic moiety (obtained by Ugi four-component reaction) through a triazole ring was developed [15] (Figure 7). The microbiological properties of the hybrid compounds and their precursors were tested against two Gram-positive (*B. subtilis* and *S. aureus*) and two Gram-negative (*E. coli* and *P. aeruginosa*) bacterial strains. It was found that the compounds inhibited Gram-negative cultures largely more than Gram-positive strains. Compound **6b** showed growth-promoting activity toward certain strains in the range of 20–200%.

**Figure 8:**
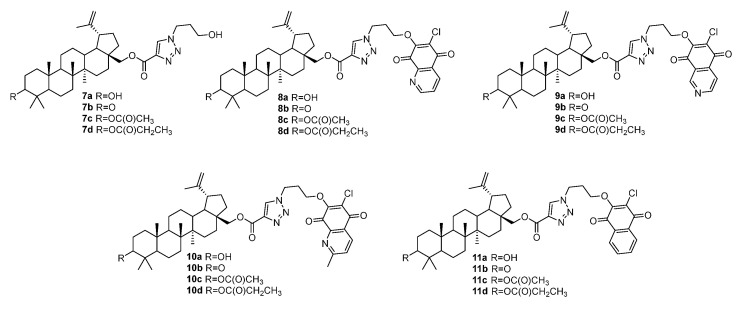
Betulin C28 1,2,3-triazole hybrids with 1,4-quinone fragment.

The 1,4-quinone scaffold was connected to betulin derivatives by the 3-hydroxypropyl-1,2,3-triazole group as a linker (Figure 8) by Professor Stanisław Boryczka and co-workers [16]. First, the C28 propynoyl betulin was obtained by treatment with propiolic acid in the presence of DCC and DMAP, then the esters were converted to triazoles **7a–7d** by a reaction with 3-azidopropanol in the presence of CuI. The reaction between the triazoles and various 1,4-quinones in the presence of K_2_CO_3_ led to hybrids **8–11**, and their anticancer activity was tested on a panel of melanoma, ovarian, breast, colon, and lung cancer cell lines. Structure–activity relationships revealed that the activity depended on the type of 1,4-quinone moiety and the tumor cell line. It was also found that the anticancer effects were enhanced in cell lines with higher levels of NQO1 protein, such as breast (T47D, MCF-7), colon (Caco-2), and lung (A549) cancers. The transcriptional activities of the genes encoding a proliferation marker (histone H3), cell cycle regulators (p53 and p21), and apoptosis pathways (BCL-2 and BAX) for selected compounds were determined. Computer modeling showed that the type of 1,4-quinone moiety influenced the location of the compound in the active site of the enzyme. It is worth noting that the study of new betulin hybrids as substrates for the NQO1 protein may lead to new medical therapeutic applications in the future. The lowest IC_50_ value (1.20 µM) was exhibited by compound **19a** in the T47D ratio, which was 2.6 times higher than the activity of cisplastin. An additional study showed that the quinone moiety reduced the lipophilicity of the resulting compounds, and their bioavailability, assessed using Lipinski and Weber’s rules, suggested the possibility of oral administration of most hybrids without signs of neurotoxicity [17].

**Figure 9:**
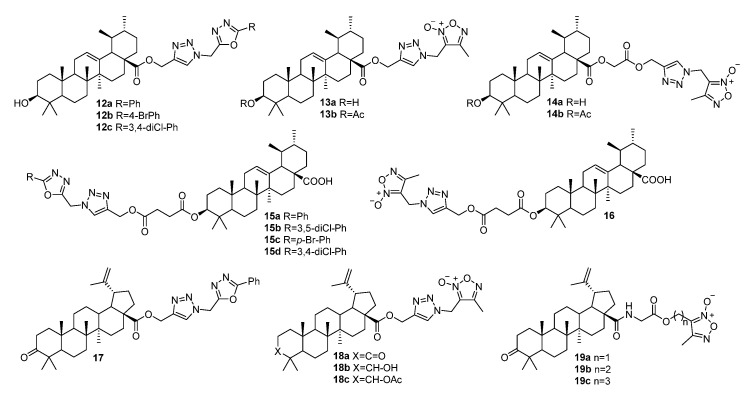
Ursolic and betulonic acid hybrids with 1,3,4- or 1,2,5-oxadiazoles and 1,2,3-triazole unit.

Ursane and lupane conjugates with oxadiazole and triazole fragments at positions C3 and C28 were obtained by the CuAAC reaction of corresponding azole-derived azides with propargyl esters [18] (Figure 9). The combination of the furoxan fragment and 1,2,3-triazole bond at the C28 position of the triterpene core was the most advantageous for the manifestation of pronounced cytotoxic activity against MCF-7 and HepG2 cells. Acetoxy-UA with methyl (1-((4-methyl-2-oxido-1,2,5-oxadiazol-3-yl)methyl)-1*H*-1,2,3-triazol-4-yl) fragment **13b** was the most active, which was superior in activity and selectivity to doxorubicin and the parent UA on MCF-7 cells. Compounds with a 1,3,4-oxadiazole ring were inactive against all cancer cells tested. The length of the carbon spacer group may be critical for cytotoxicity. Introducing an additional ester linker between the C28 carbon and the triazole ring, or changing the triazole spacer between the furoxan moiety and the triterpene backbone, resulted in decreased activity in all cells tested. In accordance with the results of molecular modeling, the activity could be explained by the interaction of new hybrid molecules and Mdm2 binding sites.

**Figure 10:**
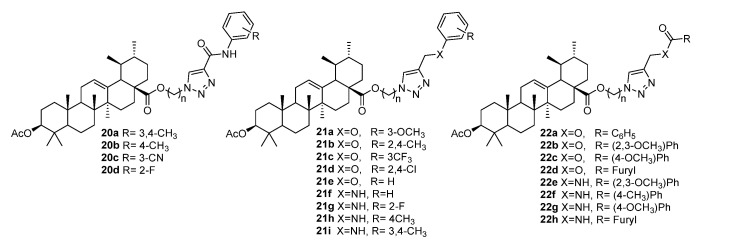
Ursolic acid C28 1,2,3-triazoles.

Another series of acetoxy-UA triazoles at C28 is presented at Figure 10. At first, the reaction with propargylbromide in the presence of K_2_CO_3_ gave the C28 propargyl ester, which was subsequently reacted with various substituted aromatic azides by the CuAAC reaction to obtain compounds **20–22** [19]. The compounds showed good PTP1B inhibitory activity (87.79–96.80%) at a concentration of 20 μg/mL and dose-dependently inhibited the PTP1B enzyme with IC_50_ values ranging from 4.15 (compound **22e**) to 14.10 μM. Molecular docking simulation and analysis suggested that compound **22e** had a good binding interaction with the active pocket.

**Figure 11:**
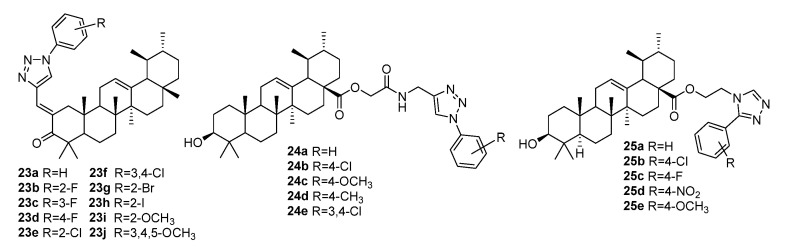
Ursolic acid C2 and C28 1,2,3-triazoles.

Ursolic acid 1,2,3-triazoles at the C2 and C28 positions were also developed as antiparasitic agents against *Toxoplasma gondii* (Figure 11) [20]. C2 derivatives **23a**–**23e** were prepared by the Claisen–Schmidt reaction with various 1-phenyl-1*H*-1,2,3-triazole-4-carbaldehydes. The triazole unit at position C28 was introduced by reacting ursolic acid with phenyl 1,2,3-triazole chloroacetamides or substituted 3-phenyl-4H-1,2,4-triazoles. Compound **25d** with a *p*-NO_2_ group exhibited the most potent in vivo activity against *T. gondii*. Determination of biochemical parameters, including liver and spleen parameters, showed that compound **25d** effectively reduced hepatotoxicity and significantly enhanced the antioxidant effect compared with UA. The molecular docking study revealed that compound **25d** had strong binding affinity to *T. gondii* calcium-dependent protein kinase 1 (TgCDPK1). Based on these data, the authors concluded that compound **25d** may act as a potential inhibitor of TgCDPK1.

**Figure 12:**
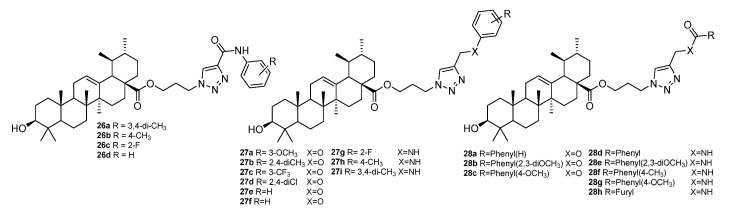
Ursolic acid C28 1,2,3-triazoles conjugated via a propane linker.

UA 1,2,3-triazoles were also tested for anti-inflammatory activity [21]. At first, UA was treated with 1,3-dibromopropane, then with NaN_3_, and the target azide was reacted with aromatic alkynes via the CuAAC reaction, giving products **26–28** (Figure 12). Compound **28b** showed a high percentage of inhibition (82.81%) compared to celecoxib (65.00%). In addition, compound **28b** was found to have strong COX-2 inhibitory activity (IC_50_ 1.16 µM, SI 64.66), similar to the COX-2 selective reference drug celecoxib (IC_50_ 0.93 µM, SI 65.47). Molecular modeling studies also indicated the most likely binding site for interactions with compound **28b** was the active site.

**Figure 13:**
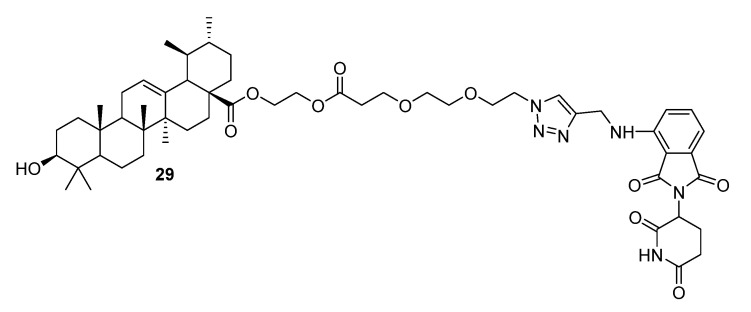
Ursolic acid PROTAC derivative.

Small-molecule proteolysis targeting chimeras (PROTACs) represent a promising approach for targeted therapy. The effectiveness of PROTACs depends on the nature of the protein/ligase ligand pair and linker. Triterpenoids also can be used as a rich POI library of PROTACs in order to improve the targeted explanation ability and drug resistance of compounds. Thus, six hybrids, applying different POE linkers, one of which contained triazole moiety **29**, were prepared (Figure 13) [22]. All compounds were assembled using DIEA/EDCI/HOBt, TEA/HATU/DMF, or inorganic bases K_2_CO_3_/KI/DMF. The reaction of UA and pre-synthesized thalidomide-POE linker units was performed to afford target compound **29**. The triazole derivative was not the most active compound; however, it exhibited promising anticancer activity with IC_50_ values of 1.89 µM, 1.87 µM, and 1.77 µM against A549, Huh7, and HepG2 cell lines, respectively.

**Figure 14:**
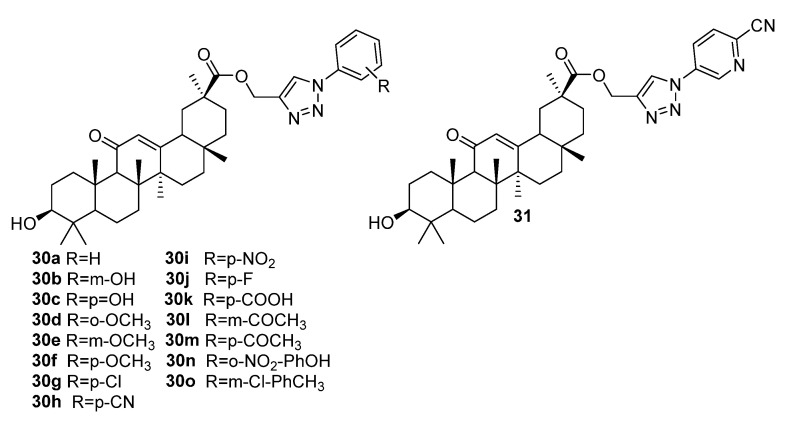
Glycyrrhetic acid C30 1,2,3-triazoles with aromatic moieties.

Glycyrrhetic acid 1,2,3-triazoles through a propargyl ester at C30 possessing various aromatic moieties were obtained and their antiplasmodial activity was studied against the chloroquine (NF-54) strain of *P. falciparum* (Figure 14) [23]. Compound **30n** was found to be the most active, with an IC_50_ value of 0.47 µM. Nevertheless, compound **17** demonstrated significant chemotherapy suppression of parasitemia and hemoglobin content, reduced liver damage, reduced inflammatory cytokine production, and reduced plasmodial infection in mouse models. The in vitro cytotoxicity/hemolysis assay suggested that compound **30n** was safe for further study of its therapeutic properties.

**Figure 15:**
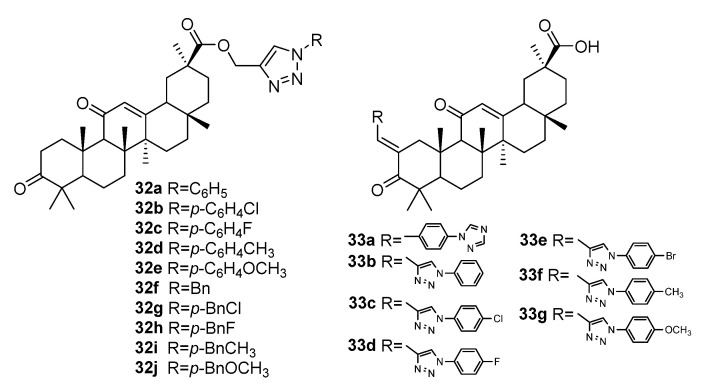
Glycyrrhetic acid-based C30 and C2 1,2,3-triazoles.

Another series of C30 GA derivatives was obtained and their anti-inflammatory activity was assessed in vitro [24]. Compounds **32a–32j** were synthesized by the CuAAC reaction of 3-oxo-glycyrrhetic acid propargyl ester with different azides (Figure 15). The introduction of a triazole unit to the C2 position (compounds **33a–33j**) occurred by Claisen–Schmidt condensation with triazolo-aldehydes. Compound **33b** suppressed the expression of pro-inflammatory cytokines, including IL-6, TNF-α, and NO, and suppressed the expression of iNOS and COX-2 in RAW264.7 cells. Western blot analysis confirmed that compound **33b** suppressed the production of proinflammatory cytokines by inhibiting the NF-κB and MAPK signaling pathways, indicating its anti-inflammatory potential.

**Figure 16:**
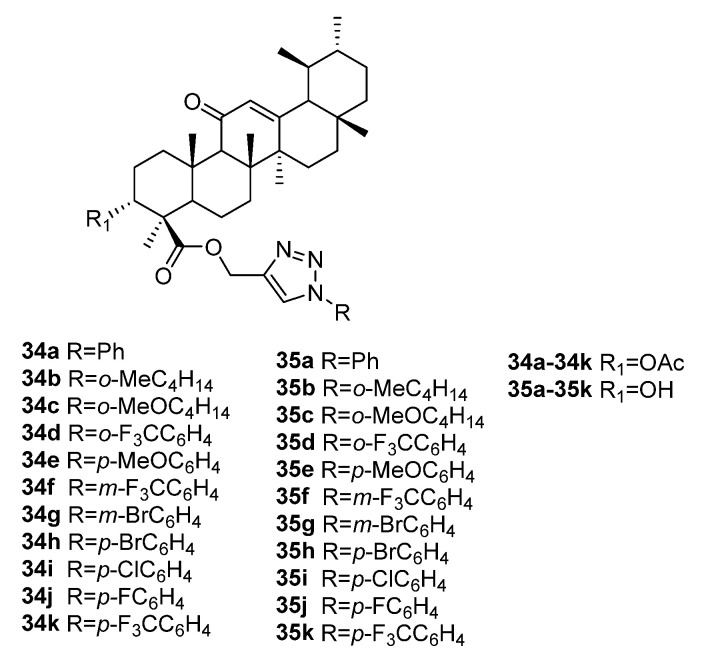
C24 1,2,3-triazoles of β-KBA.

Professor Csuk et al. used β-KBA for the synthesis of C28 and C3 triazolyl derivatives. Based on the C24 propargyl ester obtained by the reaction of β-KBA with propargyl bromide in the presence of K_2_CO_3_, a series of 22 new 1*H*-1,2,3-triazoles was obtained by the CuAAC reaction with 11 different aromatic azides and assessed for α-glucosidase inhibitory activity in vitro [25] (Figure 16). All synthesized derivatives were potent inhibitors, with IC_50_ values ranging from 0.22 to 5.32 μM. Compounds **34c**, **34f**, **34g**, **34h**, **34j**, **34k**, **35g**, **35h**, and **35k** showed exceptional inhibitory activity and were several times more potent than the parent β-KBA as well as standard acarbose. Kinetic studies showed competitive (**34f**) and mixed (**35h**) types of inhibition, with K_i_ values of 0.84 and 1.18 μM, respectively. Molecular docking studies showed that all compounds fit well into the active site of α-glucosidase, where His280, Gln279, Asp215, His351, Arg442, and Arg315 mainly stabilized the binding of these compounds. This research demonstrated the utility of incorporating a 1*H*-1,2,3-triazole moiety into the backbone of medically interesting boswellic acids. The synthesis of C3 derivatives is shown in the appropriate part (3.2. Modifications at C3 Positions).

Unlike the esters mentioned above, which contain the triazole ring substituted with different aryls, 1,4-quinone, oxadiazoles etc., C28 amides are mainly represented by complexes with cyclodextrins or sugars.

**Figure 17:**
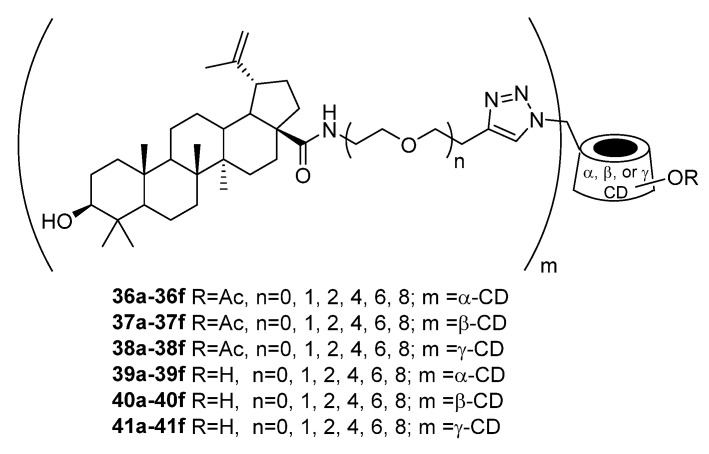
Betulinic acid cyclodextrin conjugates spacered via 1,2,3-triazole.

Professor’s Xiao research was aimed at the synthesis of conjugates of triterpene acids with cyclodextrins [26,27]. Multivalent betulinic acid derivatives **36–41** conjugated to cyclodextrins via different linkers were synthesized by the microwave CuAAC reaction and tested for activity against influenza A (H1N1) virus in vitro (Figure 17). Some compounds (**37b**, **40f**, and **41b**) exhibited moderate cytotoxicity, with viabilities ranging from 64 to 68% at a high concentration of 100 µM, while four conjugates (**36a** and **39a–39c**) effectively inhibited viral infection. The results indicated the potential of multivalent betulonic acid derivatives as antiviral agents.

**Figure 18:**
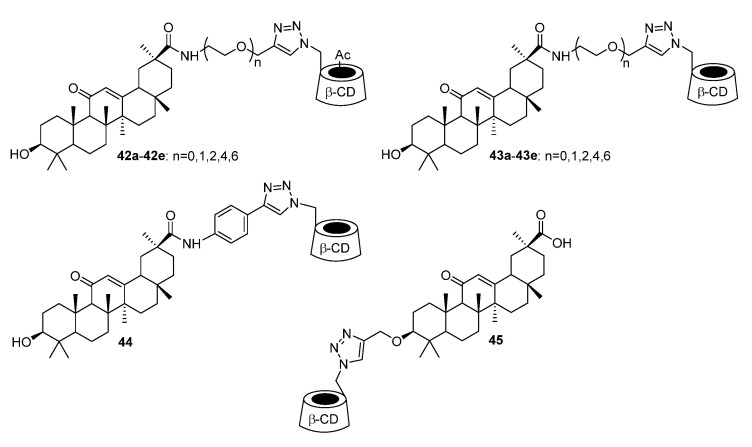
Glycyrrhetic acid β-cyclodextrin conjugates with 1,2,3-triazole spacer.

A series of water-soluble GA conjugates covalently linked to β-CD through a 1,2,3-triazole and various linkers was obtained in [28] (Figure 18). Due to the β-CD moiety, all conjugates showed lower hydrophobicity compared to the parent acid. Conjugates **42c**, **43a**, **43c**, **44**, and **45** showed promising antiviral activity against the A/WSN/33(H1N1) virus with no cytotoxicity. Among conjugates **43a–43e**, where the length of the oligoethylene glycol linker varied from 0 to 6, compound **43c** had the highest activity. Lengthening or shortening the diethylene glycol chain led to a decrease in antiviral activity. Similar results were observed for conjugates **42a–42e**, in which β-CD was acetylated. Compound **42c**, with a diethylene glycol linker, exhibited the greatest antiviral activity. The introduction of an aromatic (1,2,3-triazol-4-yl)phenyl linker maintained the moderate antiviral activity of compound **44**. Carboxylic acid derivative **45**, with a β-CD moiety at the C3 position, had the highest antiviral activity among the synthesized compounds.

**Figure 19:**
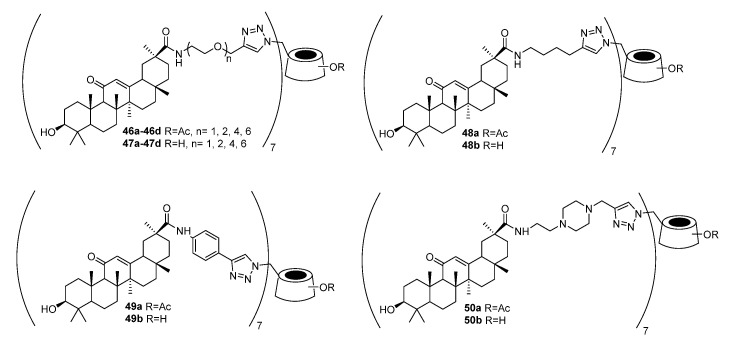
Glycyrrhetic acid β-cyclodextrin heptavalent conjugates with 1,2,3-triazole unit.

A series of GA derivatives was synthesized as potential inhibitors of influenza virus penetration (Figure 19). The synthesis strategy was based on the combination of the alkynyl-derivative with hepta-azide-substituted β-CD via the CuAAC reaction [29]. Seven conjugates demonstrated sufficient inhibitory activity against influenza virus based on the cytopathic effect reduction assay, with IC_50_ values in the micromolar range. The interaction of the most potent conjugate **50a** (IC_50_ 2.86 μM, CC_50_ >100 μM) with influenza virus was studied using the hemagglutination inhibition test. In addition, surface plasmon resonance analysis further confirmed that compound **50a** specifically bound to the HA protein of influenza virus, with a dissociation constant of 5.15 × 10^−7^ M. These results further confirmed the promising role of β-cyclodextrin as a scaffold for the preparation of various multivalent compounds as influenza penetration inhibitors.

**Figure 20:**
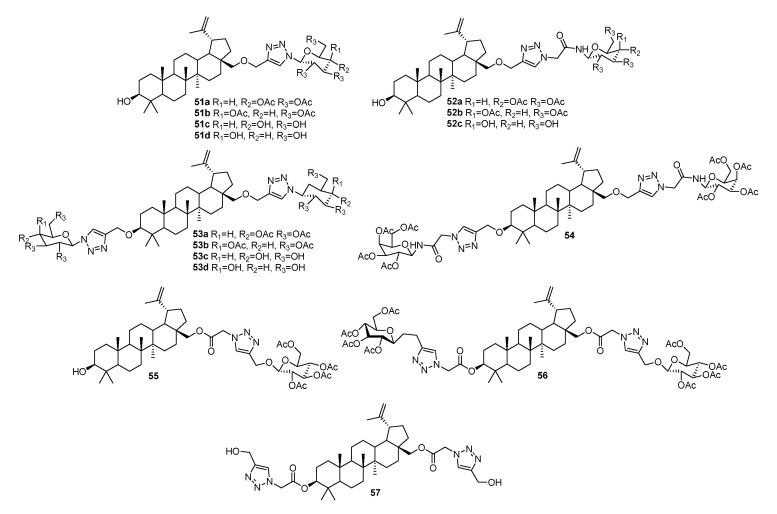
Betulin sugar mono- and di-substituted conjugates.

New betulin mono- and di-substituted derivatives with a monosaccharide through the 1,2,3-triazole ring as a linker were synthesized (Figure 20) [30]. 28-*O*-Azidoacetylbetulin or 3,28-*O*,*O*’-di(azidoacetyl)betulin, obtained by the reaction of betulin with Cl(CO)CH_2_Cl, and then with NaN_3_, were used in the synthesis of triazoles **55**–**57**. The synthesized betulin glycoconjugates were tested for cytotoxicity against human breast (MCF-7) and colorectal (HCT 116) cancer cell lines, and the results demonstrated weak antitumor activity.

**Figure 21:**
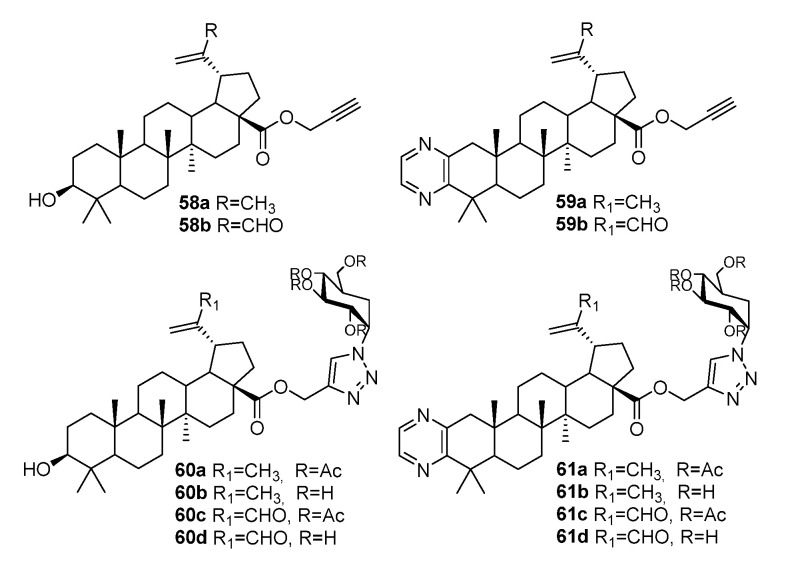
Lupane pyrazines spacered with glucose by 1,2,3-triazole ring.

A series of derivatives, including triterpenoid pyrazines, was synthesized from 3-oxo-BA using the CuAAC reaction and evaluated for cytotoxicity against 8 cancer and 2 non-cancerous cell lines [31]. The synthesis of glucose conjugates **60–61** was carried out from the corresponding triterpenic propargyl esters (**58** or **59**) by the reaction with 2,3,4,6-tetra-*O*-acetyl-β-D-glucopyranosyl azide or β-D-glucopyranosyl azide (Figure 21). The structure–activity analysis revealed that the triterpene core was responsible for the activity, while the pyrazine ring enhanced it and affected the specificity. Some derivatives (**60c** and **61c**) were broadly active (IC_50_ values in all cancer cell lines of 0.60–1.6 μM and 0.75–2.7 μM, respectively) but non-selective, while others (**59b**, **60d**, and **61d**) showed selective activity against CCRF-CEM cells (IC_50_ values of 0.43–4.6 μM).

**Figure 22:**
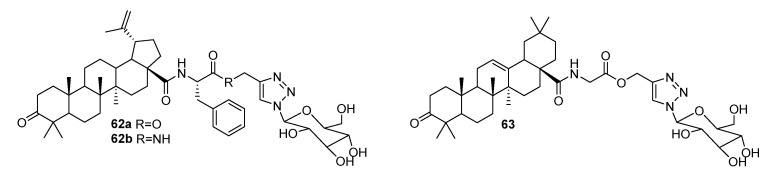
Betulonic and oleanonic acid hybrids with L-amino-acid, 1,2,3-triazole, and sugar units.

The synthesis of triterpenoids with a sugar and triazole fragments introduced through an amino acid linker was described (Figure 22) [32]. By conjugation of 3-oxo-BA or 3-oxo-OA with glycine or *L*-phenylalanine and then with propargylamine or propargyl alcohol, followed by 1,3-dipolar cycloaddition of β-D-glucopyranosyl azide, hybrid molecules **62**–**63** were obtained (no biological data are available).

**Figure 23:**
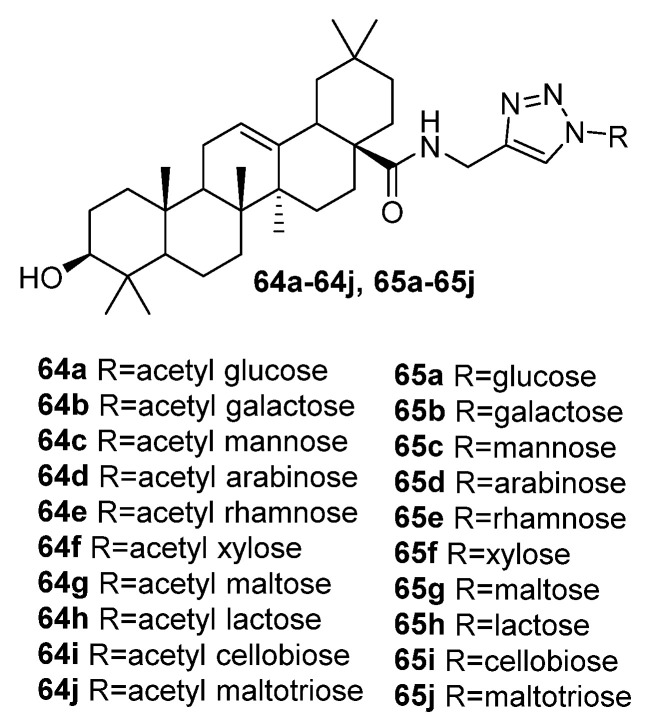
Oleanolic acid C28 *saccharide* spacered via 1,2,3-triazole.

OA saccharide conjugates were synthesized and studied for anti-influenza activity (hemagglutinin inhibition) [33]. The CuAAC reaction of various sugar azides with OA-propargylamide gave triazoles **64–65** (Figure 23). Among them, compound **65a** with a glucose moiety showed high anti-influenza activity, with an IC_50_ value of 5.47 μM, with no obvious cytotoxic effect on MDCK cells. The inhibition assay and molecular docking showed that **65a** acted on the hemagglutinin protein. Moreover, compound **65a** was active at low micromolar levels against five different strains, including influenza A and B viruses.

**Figure 24:**
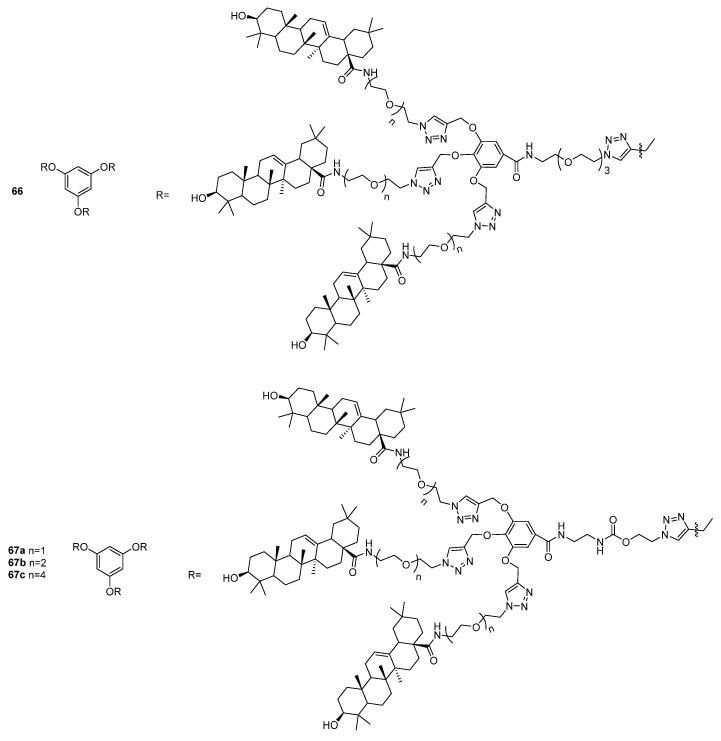
Nine-valent oleanolic acid 1,2,3-triazoles.

A series of OA nonamers (**66** and **67**), differing in linkers and structural centers, was synthesized via the CuAAC reaction and tested for antiviral activity against influenza A and B strains (Figure 24) [34]. Compounds **66** and **67a** exhibited the highest potency against influenza A (IC_50_ values of 5.23 μM and 7.93 μM, respectively), suggesting that optimal linker length was crucial for disrupting the HA–SA interaction and blocking viral recognition. Additionally, compounds **15** and **16** showed activity against an oseltamivir-resistant influenza B strain.

**Figure 25:**
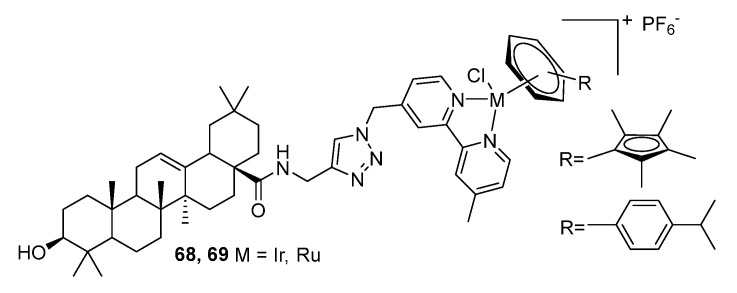
Oleanolic acid iridium and ruthenium arene complexes via 1,2,3-triazole.

Two mitochondria-targeted metal complexes Ir and Ru modified with OA through an efficient CuAAC reaction were developed [35] (Figure 25). The synthesis was carried out in two steps by the traditional amidation reaction between OA with propargylamine, followed by the CuAAC reaction of the obtained C28 alkynylamide with the metal precursors Ir-N_3_ and Ru-N_3_, which were obtained by coordination between 4-azidomethyl-4′-methyl-2,2′-bipyridine and Ir/Ru species. The OA-Ir and OA-Ru complexes were highly cytotoxic to A2780 cells, causing mitochondrial destruction, including decreased mitochondrial membrane potential, inhibition of mitochondrial respiration, and decreased intracellular ATP levels, as well as inducing autophagy. In addition, OA-Ir and OA-Ru effectively suppressed the metastasis of ovarian cancer cells through the mechanism of suppressing MMP2/MMP9 and disrupting F-actin dynamics. It is worth noting that OA-Ir and OA-Ru could also efficiently degrade 3D multicellular tumor spheroids, demonstrating potential for clinical application.

**Figure 26:**
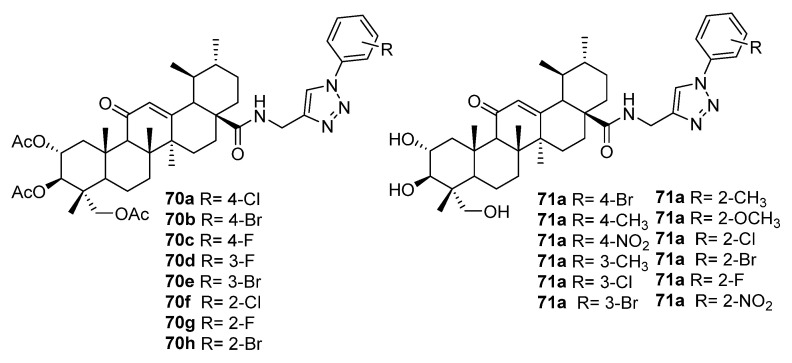
Asiatic acid C28 1,2,3-triazoles.

A series of 1,2,3-triazole derivatives (**70** and **71**) based on AA was obtained [36] (Figure 26). The triazole ring was introduced by the reaction of acylated 11-oxo-AA with propargyl amine in the presence NaH, followed by treatment with different substituted aromatic azides under CuAAC reaction conditions. Compound **71a** showed inhibitory activity against the A549 cell line, with an IC_50_ value of 2.67 μM. Molecular docking studies revealed key interactions of **71a** with NF-κB, in which the 1,2,3-triazole moiety and the hydroxyl groups of the AA backbone were important for improving the inhibitory activity. Mechanism of action studies demonstrated that the antitumor effect was achieved by inducing apoptosis and inhibiting cell migration in vitro.

**Figure 27:**
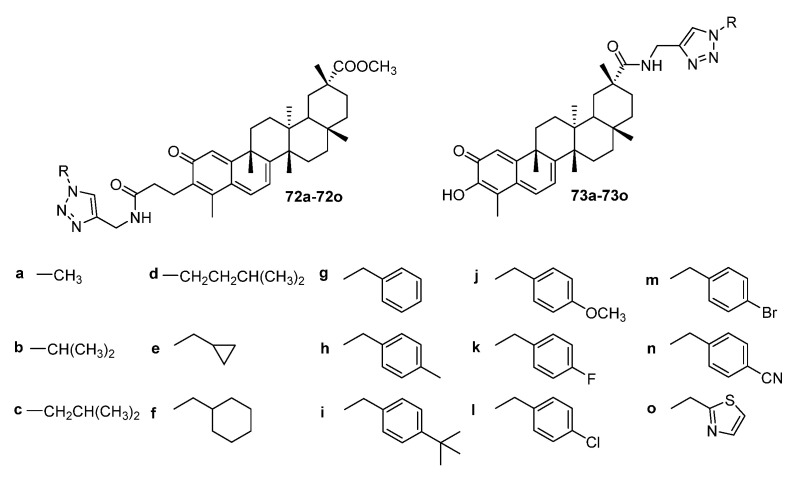
Celastrol C3 and C30 1,2,3-triazoles.

In search of effective drugs against glioma, a series of celastrol derivatives containing a 1,2,3-triazole moiety linked via an amide group (**72** and **73**) was synthesized (Figure 27) [37]. The triple bond was introduced in the C3 position through the formation of a C3 carboxymethoxyether and further propargylation of the carboxyl group. The reaction with 2-aminopropyne in the presence of HATU and DIPEA gave celastrol propargylamide. By the CuAAC reaction of the obtained alkynes, target triazoles **72** and **73** were prepared. The activity against four human glioma cell lines (A172, LN229, U87, and U251) was assessed using an in vitro MTT assay. The results showed that compound **73i** (IC_50_ value of 0.94 µM) exhibited significant antiproliferative activity against the U251 cell line, which was 4.7 times greater than that of the parent celastrol (IC_50_ value of 4.43 µM). In addition, compound **73i** significantly inhibited colony formation and migration of U251 cells.

**Figure 28:**
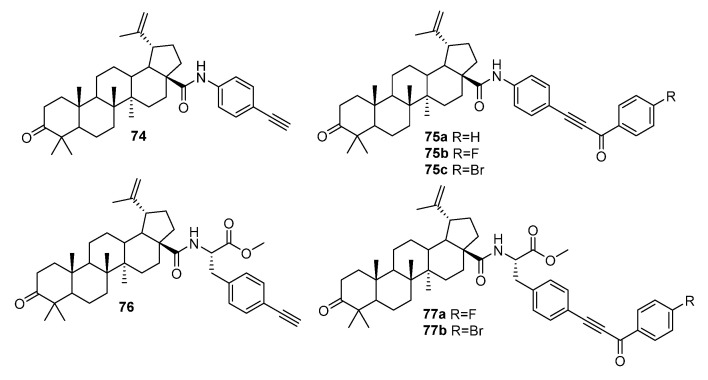
Betulonic acid arylpyrimidine amide alkynyl ketones.

New lupane alkynyl-triterpenoids were prepared with the cross-coupling reaction of alkynes **74** and **76** with benzoic acid chlorides, affording alkynyl ketones **75** and **77** (Figure 28) [38]. The compounds were the intermediates for the synthesis of new arylpyrimidine betulonic acid amide conjugates, which exhibited significant and selective anti-inflammatory activity.

**Figure 29:**
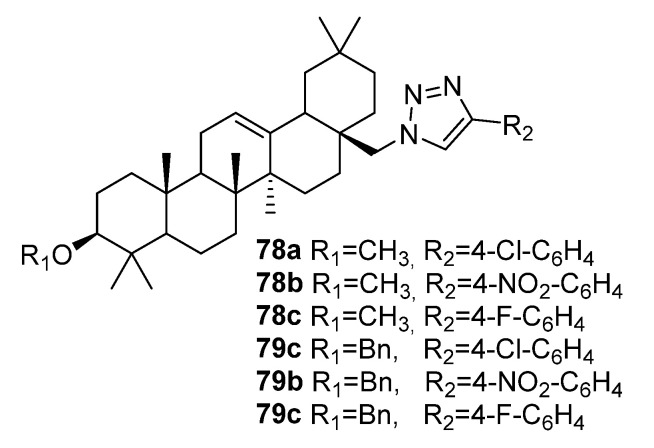
OA conjugates with 1,2,3-triazole ring at C28.

In order to develop a potential bactericidal inhibitor of glucosamine-6-phosphate synthase (GlmS), a series of oleanane-type derivatives with a triazole moiety was synthesized in some steps, including the reduction of the carboxyl group of **OA** to an alcohol group, followed by treatment with trifluoromethanesulfonic anhydride, and finally with NaN_3_ (Figure 29) [39]. The obtained C28 azide reacted with substituted phenylacetylene or alkyl alkyne, leading to target triazoles **78** and **79**. The conjugates were active against six pathogenic fungi at a concentration of 50 μg/mL, showing significant inhibitory effects against Sclerotinia sclerotiorum, Botrytis cinerea Pers, and Rhizoctonia solani Kuhn, with inhibition rates above 50%. Compared with the parent OA, the antifungal activity was significantly improved. The antifungal effects of compounds **78a–78c** and **79a–79c** against *S. sclerotiorum* were particularly remarkable, at 85.6%, 83.1%, 87.6%, 86.8%, 87.7%, and 89.6%, respectively. Structural analysis of the compounds revealed that the benzene ring (e.g., fluorine, chlorine, nitro) electron-withdrawing substituent increased the activity.

### 3.2. Modifications at C3 Positions

**Figure 30:**
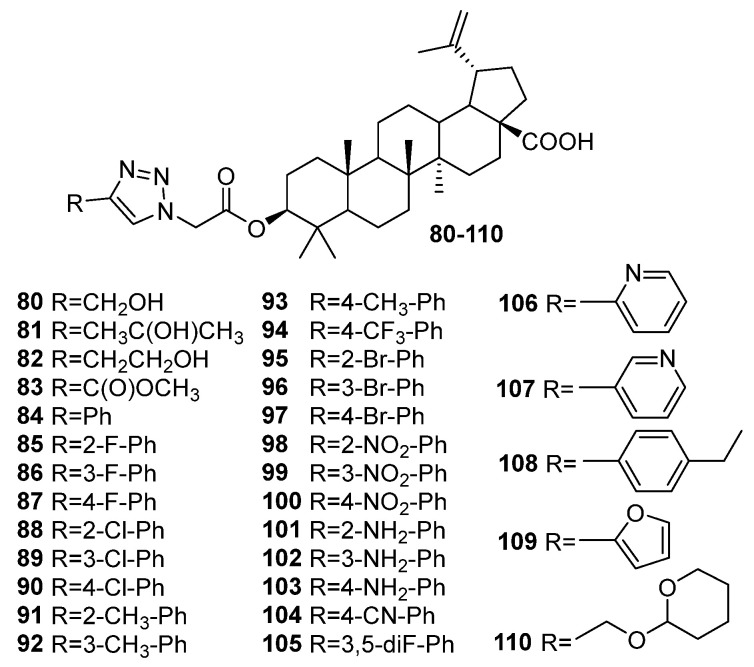
Betulinic acid C3 1,2,3-triazoles.

A series of betulinic acid 1,2,3-triazoles (**80–110**) was synthesized as α-glucosidase inhibitors with hypoglycemic activity (Figure 30) [40]. All of the derivatives exhibited excellent inhibition against α-glucosidase, while compound **102**, with a 3-amino-phenyl group in the triazole ring, was the most active (IC_50_ value of 2.83 μM), being a mixed-type inhibitor. According to in vivo experiments, compound **102** reduced the level of fasting blood glucose and improved glucose tolerance and dyslipidemia.

**Figure 31:**
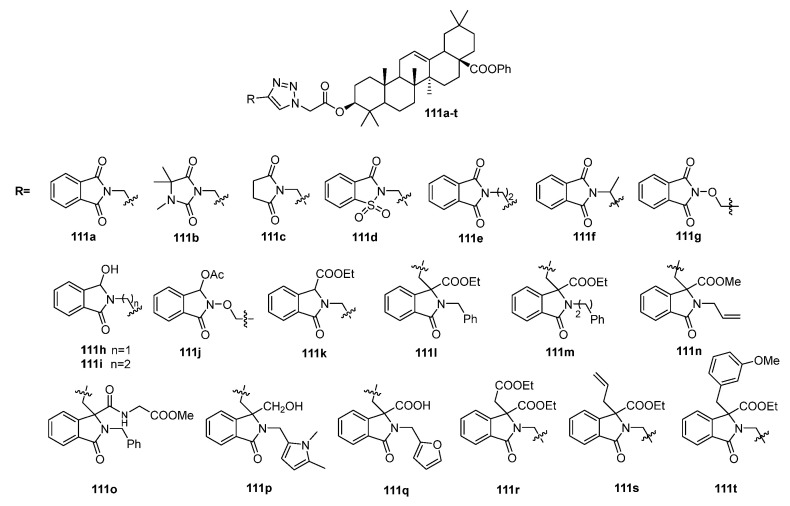
Oleanolic acid C3 phthalimidine conjugates via 1,2,3-triazole unit.

A series of structurally novel OA-phthalimidines (isoindolinones) (**111a–t**) with 1,2,3-triazole moieties was synthesized and evaluated for activity against Gram-positive and Gram-negative bacteria (Figure 31) [41]. The synthetic route included the reaction of **OA** with ClCH_2_COCl and further treatment with NaN_3_ to give C3-azidoacetyl-OA, which reacted with propargylated phthalimidines using the CuAAC reaction approach. Derivatives **111a–t** were screened against Gram-positive bacteria (*S. aureus* and *L. monocytogenes*) and Gram-negative bacteria (*S. thyphimurium* and *P. aeruginosa*). Compared to the parent **OA**, compounds **111d**, **111g**, and **111h** displayed the strongest antibacterial effect against *Listeria monocytogenes*. Molecular docking studies, which investigated how the most potent derivatives bound to the active site of the Lmo0181 ABC substrate-binding protein in L. monocytogenes, revealed that both hydrogen bonding and hydrophobic interactions were crucial for activity.

**Figure 32:**
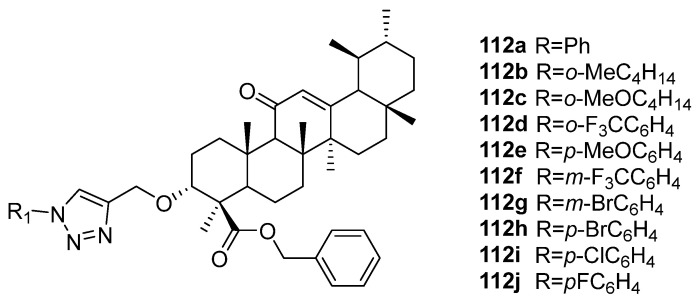
Boswellic acid C3 1,2,3-triazoles.

A series of derivatives of β-KBA containing a 1,2,3-triazolyl ring (**112a–112j**) at the C3 position was obtained from β-acetoxy-KBA in four steps: benzyl protection of the carboxylic group, deacetylation, esterification of the C3 hydroxyl group using propargyl bromide in the presence of NaH, and the CuAAC reaction with various azides (Figure 32) [42]. The cytotoxic activity was studied against triple negative breast cancer cells (MDA-MB-231) and normal (MCF-10A) cells. In addition, all of the synthesized derivatives exhibited strong antiproliferative activity, with IC_50_ values ranging from 4.45 μM to 14.45 μM. Among them, compounds **112j**, **112f**, and **112i** exhibited exceptional inhibitory activity and were found to be several times more potent than the parent compounds. In addition, the drug targets and mechanisms of action were established using chemoinformatics and molecular docking. The results demonstrated that these compounds could target CHK1 to induce anticancer effects in TNBC.

**Figure 33:**
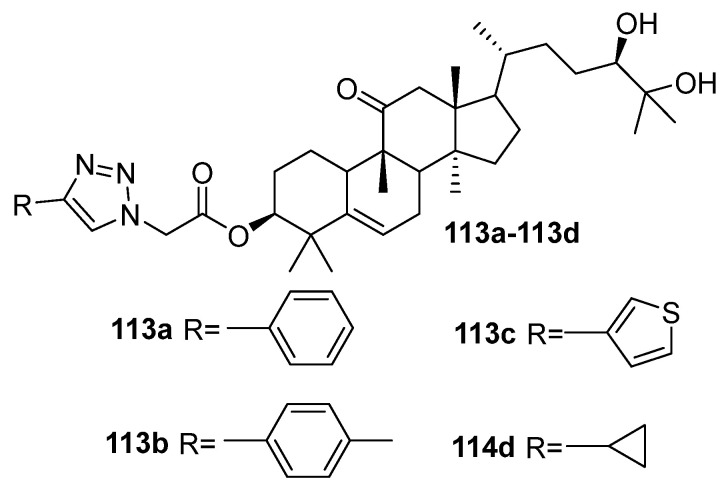
Mogrol C3 1,2,3-triazoles.

A cucurbitane-type tetracyclic triterpene mogrol was used for alkyne modification at the C3 OH position [43]. The C3 hydroxyl group first reacted with 2-azidoacetic acid to afford the 2-azidoacetate derivative, then the CuAAC reaction led to derivatives **113a–113d** (Figure 33). The compounds were tested for activity against A549, NCIH460, and CNE1 cancer cell lines. The results obtained demonstrated improved cytotoxicity for the modified analogs. For example, compound **M6c** was found to be ten times more active against NCI-H460 cells (IC_50_ value of 10.59 μM) than parent mogrol.

**Figure 34:**
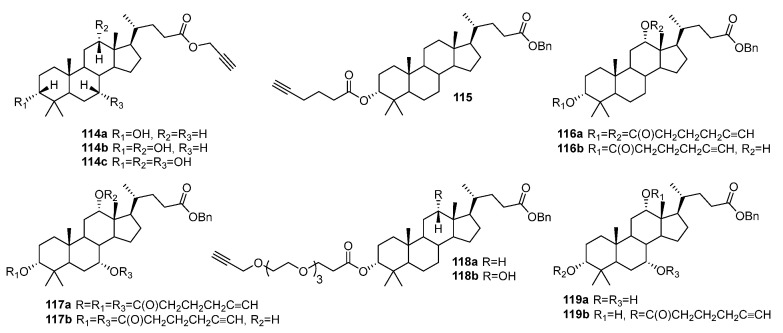
Bile acid acetylenic derivatives.

Alkynyl steroids are also the focus of intensive research. Three groups of bile acid derivatives with acetylenic moieties, such as propargyl, hex-5-ynoyl, and 4,7,10,13-tetraoxahexadec-15-ynoyl ones, were obtained (Figure 34) [44]. Propargyl derivatives **114a–114c** were synthesized by the reaction of bile acids with propargyl bromide in the presence of K_2_CO_3_. Other alkynes (**115–119**) were synthesized using 5-hexynoic or 4,7,10,13-tetraoxahexadec-15-ynoic acids under the action of EDCCL and DIPEA. The cytotoxic activity against hepatocellular carcinoma (HepG2, Huh7), prostate cancer (PC3), and human embryonic kidney (HEK293) cell lines was studied. As can be seen, the derivatives are arranged in the following order according to their cytotoxic activity: **115**–**117** < **118**–**119**. Complete blocking of the hydrophilic groups of bile acids by hydrophobic fragments (benzyl and hex-5-inol) led to the loss of cytotoxicity. In turn, the literature notes that the cytotoxicity of the modified FAs depended on their hydrophobicity, charge, and degree of hydration.

**Figure 35:**
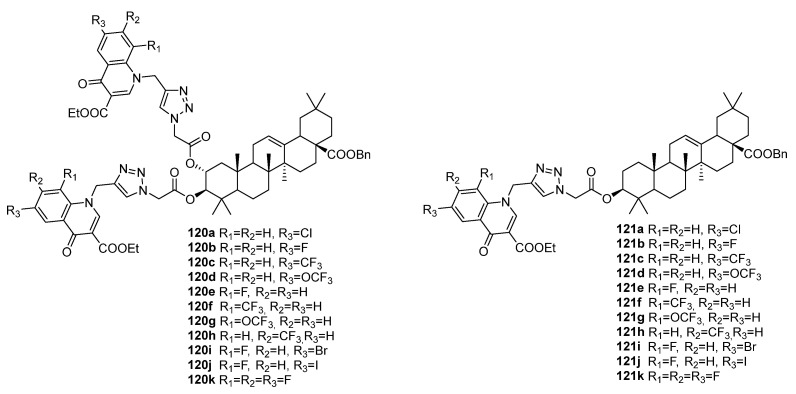
Maslinic C2, C3 and oleanolic acid C3 1,2,3-triazoles with quinolone moieties.

A series of **MA** and **OA** triazole hybrids possessing quinolone moieties with potent antibacterial and antibiofilm activities was synthesized and their antibacterial and antibiofilm activities were evaluated (Figure 35) [45]. It was found that 2,3-bis-quinolone conjugates were more potent than C3 mono-analogs. Compounds **120b**, **120c**, **120f**, and **120j** exhibited significant antibacterial activity against both Gram-positive and Gram-negative bacteria when compared to their analogs and the reference drugs.

**Figure 36:**
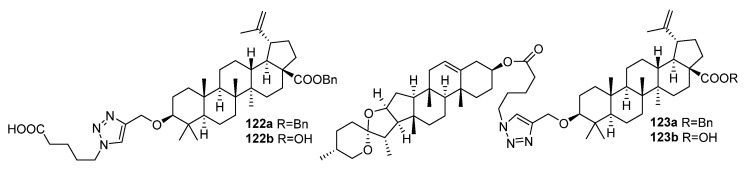
Betulinic acid-diosgenin conjugates with 1,2,3-triazole link.

Betulinic acid-diosgenin conjugates (Figure 36) via the triazole unit with cytotoxic activity are described in [46]. 3-*O*-propargylated BA reacted with azidovaleric acid under CuAAC conditions, affording compounds 122a and 122b. Conjugation of triazole 122a with diosgenin by the DCC method with further deprotection gave compounds 123a and 123b. Conjugate 123a showed selective cytotoxicity against human T-lymphoblastic leukemia (CLE) cancer cells (IC_50_ value of 6.5 µM), in contrast to conjugate 123b, which did not exhibit cytotoxicity, and parent diosgenin, an adaptogen that has the potential to act on the central nervous system. Compound 122a demonstrated moderate multivariate cytotoxicity in human T-lymphoblastic leukemia (CEM), human cervical cancer (HeLa), and human colon cancer (HCT 116) cells. BA and alkyne intermediates did not exhibit cytotoxicity in the cancer cell lines tested.

**Figure 37:**
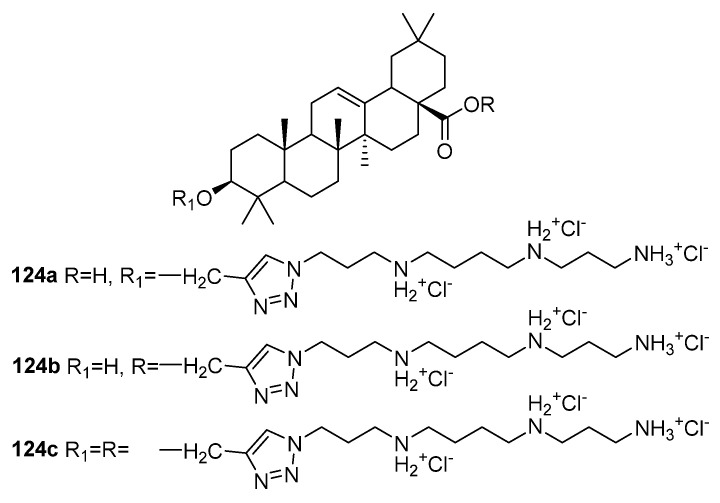
Oleanolic acid C3 and C28 1,2,3-triazoles with spermine moieties.

Self-assembly of natural compounds is a relatively new but promising direction in the chemistry of triterpenoids. An important contribution in this field was made by Professor Wimmer and co-workers, who prepared triazoles with long-chain amines and considered them as nanomolecules that self-organized in water. **OA**-based 1,4-disubstituted 1,2,3-triazole linkers with one or two spermine moieties were prepared through mono- or di-propargylated **OA** by the CuAAC reaction with azido-spermine (Figure 37) [47,48]. Conjugates **124a** and **124b** were studied by means of cryogenic transmission electron microscopy (cryo-TEM), showing self-assembly into kinetically preferred metastable micellar nanoparticles (d ≈ 6–10 nm) and then into thermodynamically stable helical nanofibers. In addition, the compounds exhibited high antimicrobial activity against a number of G+ bacteria (MIC 1.13–2.26 μg mL^−1^), suggesting that they may become important multifaceted supramolecular nanostructures that will be useful as selective antimicrobial agents.

**Figure 38:**
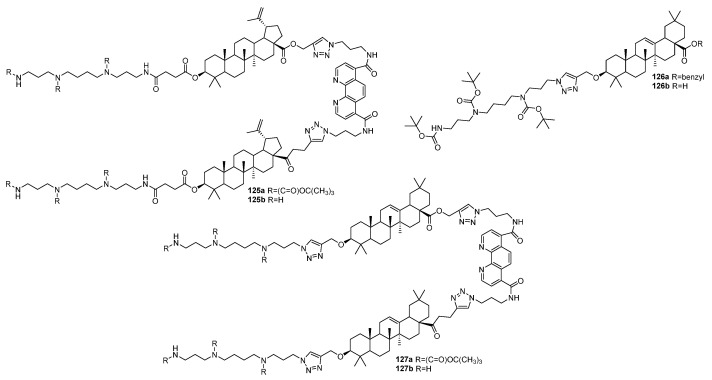
Betulinic and oleanolic acids1,2,3-triazoles with spermine and 1,10-phenanthroline fragments.

Another series of new amphiphiles of lupane and oleanane types possessing spermine and additional 1,10-phenanthroline units spaced through the triazole ring at the C3 position is also described [49] (Figure 38). The C3 OH group of betulinic acid was esterified with succinic anhydride to obtain the hemisuccinate ester, which was subjected to a condensation reaction with Boc-protected spermine, while the C17 COOH group was transformed to a propargyl ester by alkylation with propargyl bromide. Next, the alkynyl derivative and substituted phenanthroline with an azide group were subjected to the CuAAC reaction, affording the complex molecules **125a** and **125b**. In **OA**, the carboxyl group was protected as a benzyl ester, and the C3 OH group was converted to the propargyl ether. The obtained molecule reacted with azido-spermine via the CuAAC reaction to form the conjugates **126a** and **126b**. The free carboxyl group in **126b** was converted to a propargyl ester and, using azido-phenanthroline reagent, **127a** and **127b** were synthesized. The target compounds self-assembled into helical or plate-shaped nanostructures depending on the structure of the target molecule and the way the spermine subunits bound to other molecules. They also proved their ability to coordinate ^64^Cu(II) ions. In addition, the target compounds exhibited antitumor activity that was partially dependent on the formation of nanostructures. Compounds **125b** and **127b**, with a free C28 carboxyl group, showed higher cytotoxicity against cancer cell lines MCF7 (IC_50_ values of 3.0 and 4.1 µM, respectively) and HeLa (IC_50_ values of 5.7 and 6.9 µM, respectively) compared to cisplatin.

**Figure 39:**
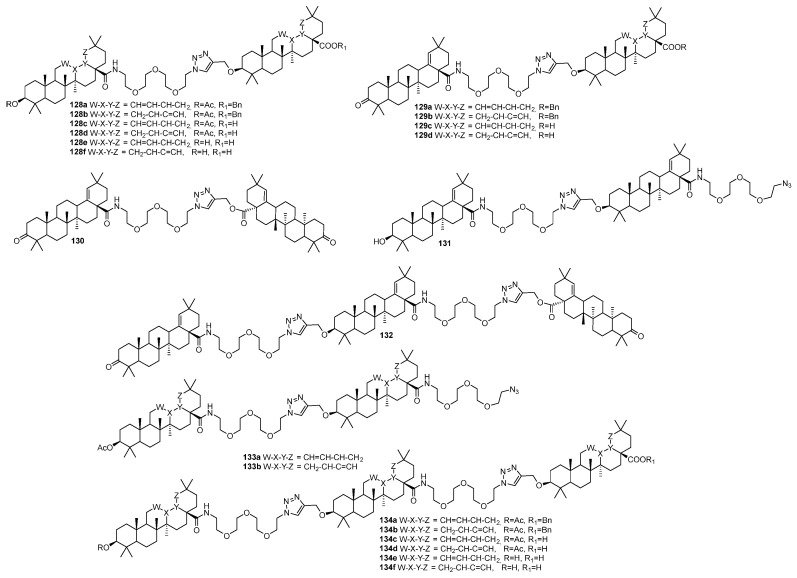
Oleanane-type triterpene–PEG ribbon conjugates with 1,2,3-triazole spacer.

The strategy of designing molecular ribbons of oleanolic, morolic, or moronic acids was described in [50] (Figure 39). Derivatives **128–134** were synthesized using a PEG3 junction, 1,2,3-triazole rings, and amide bonds between triterpenic molecules and were screened for antiviral, antimicrobial, and cytotoxic activities. Compound **128e** showed activity against HIV-1 (EC_50_ 50.6 µM), while **129d** exhibited cytotoxicity toward melanoma cells (G-361; IC_50_ value of 20.0 µM). Several compounds displayed antimicrobial effects, with **128e** inhibiting *S. aureus* and *E. coli*, **134f** inhibiting *S. aureus*, and **130** inhibiting *E. faecalis* (>50% (c 25 µg·mL^−1^), >40% (c 25 µg·mL^−1^), and >62% (c 25 µg·mL^−1^), respectively). Furthermore, compounds **130**, **129c**, and **129d** formed gels in glycerol, and **128e** formed a gel in ethylene glycol, indicating supramolecular gel-forming ability.

### 3.3. Triazoles Fused with the A-Ring

**Figure 40:**
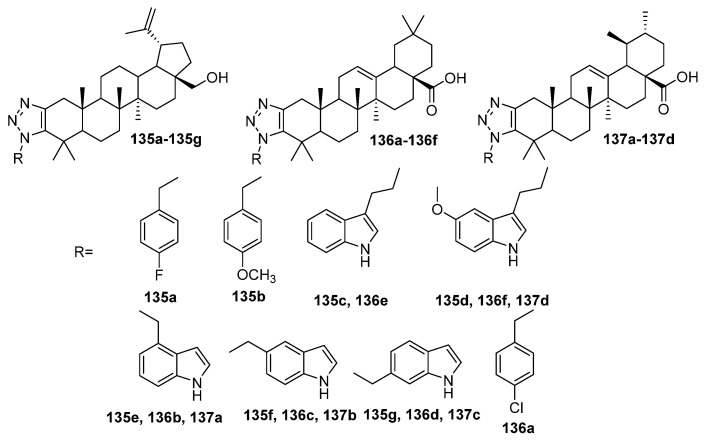
1,2,3-Triazole-fused triterpenes of lupane, oleanane, and ursane types.

Professor Dehaen’s group reported the synthesis of new 1,2,3-triazole-fused triterpenes [51]. As starting materials, they used **BE**, **OA**, and **UA**, which were involved in a multi-component triazolization reaction with 4-nitrophenyl azide and various amines, leading to A-ring-fused 1,2,3-triazoles **135–137** (Figure 40). The obtained derivatives were investigated for antitumor activity in twelve cancer cell lines and for antibacterial activity. **BE** derivatives **135a–135g** showed enhanced antibacterial activity against *E. coli* and *S. enterica* compared to **BE**. Compounds **125c** and **135d** were the most potent *S. enterica* inhibitors (MIC_50_ ~17 mg/mL) and also active against *E. coli* (MIC_50_ values of 20.92 mg/mL and 17.71 mg/mL, respectively. Oleanolic acid derivatives **136a–136d** exhibited improved activity against Gram-negative bacteria but reduced activity against Gram-positive bacteria, while compounds **136e–136f** were inactive against Gram-negative bacteria. Ursolic acid derivatives **137a–137d** were less active than **UA** against Gram-positive bacteria and inactive against Gram-negative bacteria. Cytotoxicity screening revealed that **136b–136f** and **137a–137d** displayed strong activity against several cancer cell lines, with **136f** showing the most potent effect against HL-60 cells (IC_50_ value of 1.3 µM) by inducing mitochondrial dysfunction, G0/G1 cell cycle arrest, apoptosis, and autophagy.

**Figure 41:**
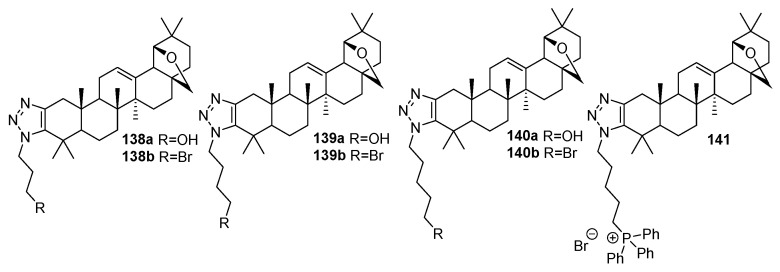
Allobetulin A-ring fused 1,2,3-triazoles.

According to the above-mentioned strategy [51], six 1,2,3-triazole derivatives of allobetulin **138–140** and one triphenylphosphonium-linked derivative **141** were prepared and their cytotoxicity toward seven cancer cells and two normal cells was tested (Figure 41) [52]. Compounds **138–140** were prepared by the method described in [51]. The target triphenylphosphonium-linked derivative of allobetulin **141** was synthesized from **140a** following reactions with PBr_3_ and PPh_3_. The cytotoxicity screening results showed that the target triphenylphosphonium-linked derivative of allobetulin **141** was the most potent and displayed excellent inhibitory effects against all cancer cell lines, with IC_50_ values ranging from 1.05 μM to 1.2 μM, and it had the same anticancer activity as the commercial anticancer drug doxorubicin but was more active than cisplatin.

### 3.4. Modifications at C30, C19, and C2 Positions

**Figure 42:**
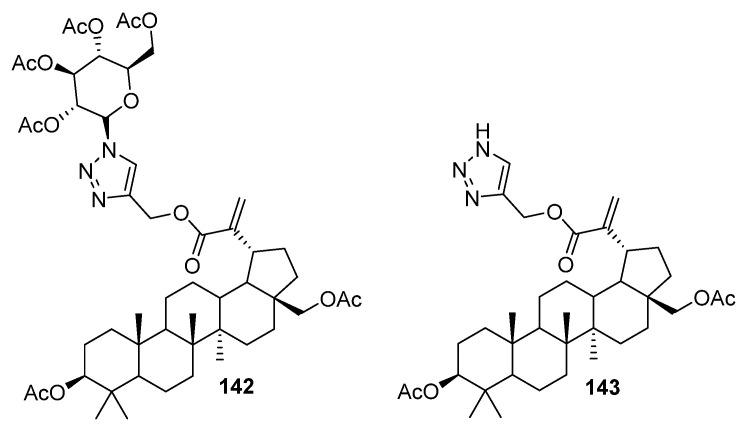
Di-acetoxy-betulin C30 1,2,3-triazoles.

On the basis of lupane, C30 propargyl ester derivatives **142** and **143** were synthesized (Figure 42) and evaluated in salsolinol (SAL)- and glutamate (Glu)-induced models of neurodegeneration in neuron-like SH-SY5Y cells [53]. It was shown that betulin triazoles **142** and **143** showed a pronounced neuroprotective effect; however, compound **143** with a free triazole ring was cytotoxic at high concentrations. Both derivatives reduced oxidative stress and caspase activity, but **143** was more effective, similar to the inhibitor Ac-DEVD-CHO. Compounds **142** as well as **143** blocked the opening of the mitochondrial permeability pore, but only **142** restored the mitochondrial membrane potential. In the Glu model, **143** more strongly reduced oxidative stress, and the neuroprotection of **142** was probably associated with the restoration of the mitochondrial membrane potential.

**Figure 43:**
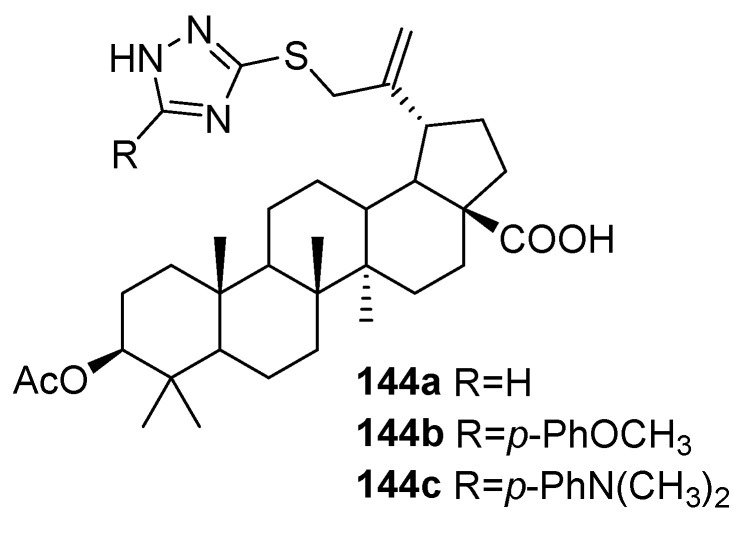
Betulinic acid C30 1,2,4-triazole-3-thiol derivatives.

Professor Soica and co-workers obtained a series of betulinic acid C30 1,2,4-triazole-3-thiol derivatives. 30-(1*H*-1,2,4-triazole-3-ylsulfanyl)-betulinic acids **144a–144c** were synthesized in two steps by the bromination of acetyl-BA with NBS, followed by reaction with the corresponding 1,2,4-triazole-3-thiol (Figure 43) [54]. The compound **144a** exhibited significant cytotoxic effects, significantly reducing cell viability compared to the control. Testing of the compound on normal HaCaT cells showed no toxicity, indicating its selective dose-dependent anticancer activity. Investigation of the underlying molecular mechanism revealed an apoptotic effect induced at the mitochondrial level, which was confirmed by high-resolution respirometry studies. BA aromatic-substituted C30 1,2,4-triazole-3-thiols **144b** and **144c** showed lower IC_50_ values in almost all cases compared to their predecessors, exhibiting the greatest cytotoxicity against the RPMI-7951 cell line (IC_50_ values of 18.8 μM for **144b** and 20.7 μM for **144c**). Both compounds inhibited mitochondrial respiration in RPMI-7951 cells, revealing a mechanism of apoptosis induced at the mitochondrial level. In addition, the derivatives caused a significant decrease in the expression of the anti-apoptotic gene Bcl-2 while increasing the expression of the pro-apoptotic gene BAX [55].

**Figure 44:**
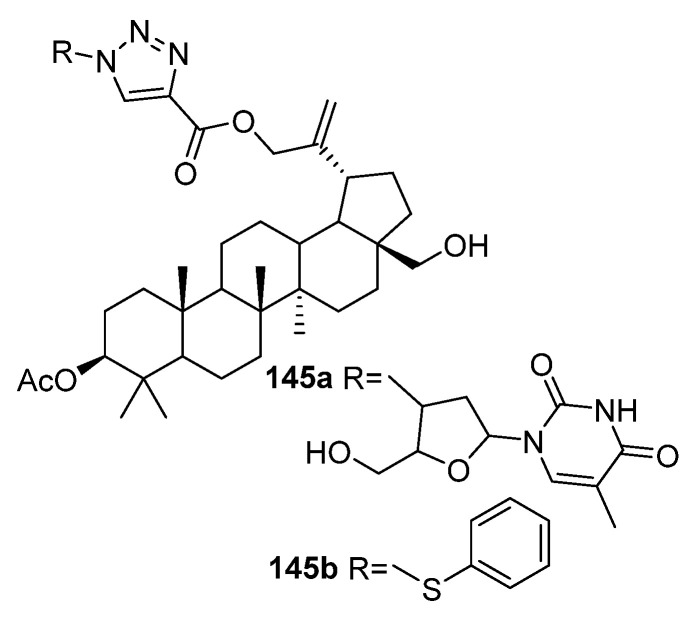
Betulin C30 1,2,3-triazoles.

Another series of C30-substituted triazolyl derivatives was synthesized by Professor Bebenek and co-workers [56]. Novel C30-substituted diacetoxybetulin derivative **145a** linked to AZT via a 1,2,3-triazole ring (Figure 44) was synthesized by the CuAAC reaction of 30-propynoylbetulin, obtained by the Steglich reaction, with azidothymidine (AZT). The cytotoxicity screening showed significant activity of this conjugate against MCF-7 cells (IC_50_ value of 4.37 µM). The use of the similar approach led to C30-substituted triazole **145b** with X-ray diffraction data confirmation of the obtained structure (Figure 44) [57].

**Figure 45:**
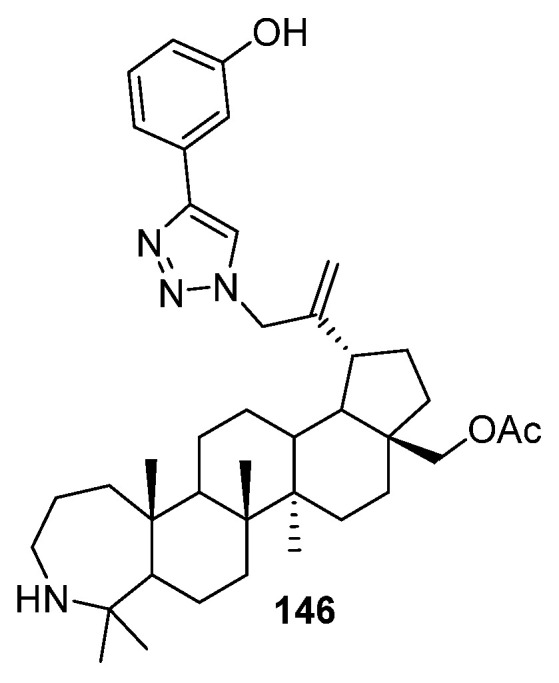
Azepanobetulin C30 1,2,3-triazole.

By successful reactions of 28-acetoxy-A-azepanobetulin with NBS and NaN_3_ and the CuAAC reaction with 3-hydroxyphenylacetylene, 30-[4-(3-hydroxyphenyl)-1*H*-1,2,3-triazol-1-yl]-3a-aza-3a-homolup-20(29)-en-28-yl acetate (**146**) was obtained (Figure 45) [58].

**Figure 46:**
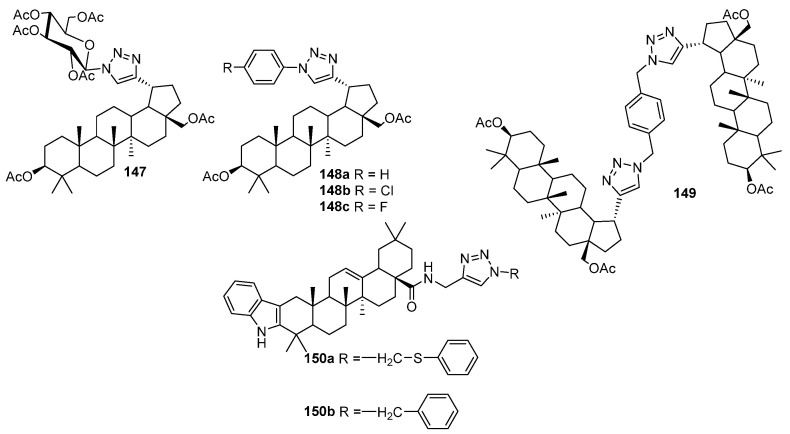
Lupane C19 and oleanane C28 1,2,3-triazoles.

A series of lupane C19-(1,2,3-triazolyl)-triterpenoids (**147–149**) was prepared by the CuAAC reaction of 29-nor-20(30)-yne betulin with different aromatic and sugar azides (Figure 46) [59]. Unlike other studies, catalysts in the form of copper salts were not effective due to the low reactivity of the hindered C19 alkyne (confirmed by DFT calculations). As a result, the most efficient catalyst was the chosen dinuclear copper complex [Cu(μ-OH)(TMEDA)]_2_Cl_2_, which also turned out to be a catalyst in the synthesis of bis(triazolyltriterpenoid)-derivative **149**. Some time later, **147–149**, as well as oleanane-type triterpenoids **150a** and **150b** (Figure 46) with the C28 1,2,3-triazolyl-group were tested for antiviral activity against HCMV, HSV-1, and HPV-11 types viruses [60]. 2,3-Indolo-OA C28 triazole with a benzyl substituent **150b** was the most active against the HCMV virus, with EC_50_ values of <0.05 (SI > 81). Lupane 3,28-diacetoxy-triazole with phenyl (**148a**) and fluorophenyl (**148c**) fragments possessed the highest activity among all screened compounds toward the HPV-11 type virus, with EC_50_ values of 2.97 μM and 1.20 μM and SI_90_ values of 28 and >125, respectively.

**Figure 47:**
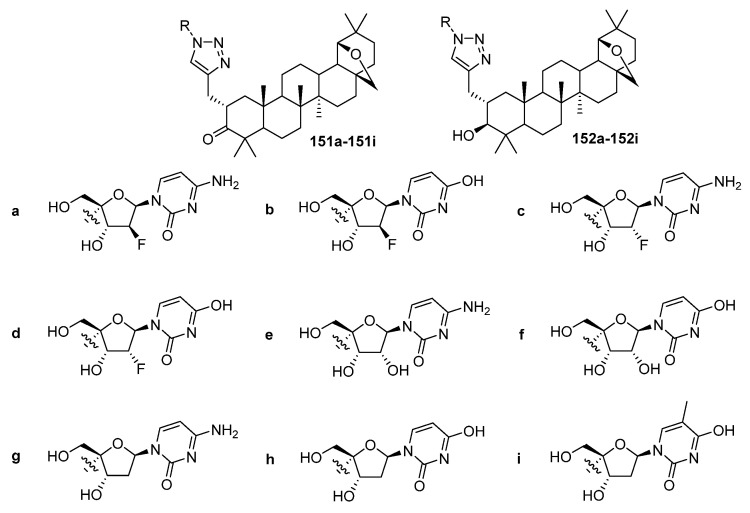
Allobetulone/allobetulin C2 nucleoside 1,2,3-triazole conjugates.

α-Alkylation of 3-oxo triterpenoids with propargyl bromide in the presence of strong bases (KN(SiMe_3_)_2_, Et_3_B, or t-BuOK) is an original approach affording C2-substituted alkynyl derivatives [7,8]. In this way, a series of allobetulone/allobetulin-nucleoside conjugates **151–152** (Figure 47) was synthesized. Among them, compounds **151b**, **151e**, **152a** and **152d** showed promising antiproliferative activity against six cell lines with zidovudine, cisplatin, and oxaliplatin as reference drugs [61]. Compound **152d** exhibited much more potent antiproliferative activity against human cancer cell lines SMMC-7721, HepG2, MNK-45, SW620, and A549 than cisplatin and oxaliplatin. In a preliminary mechanism of action study, compound **152d** induced cell apoptosis and autophagy in SMMC cells, leading to antiproliferation and G0/G1 cell cycle arrest by regulating the protein expression levels of Bax, Bcl-2, and LC3. The results indicated that nucleoside replacement had a positive effect on improving the antitumor activity of allobetulin/allobetulone.

**Figure 48:**
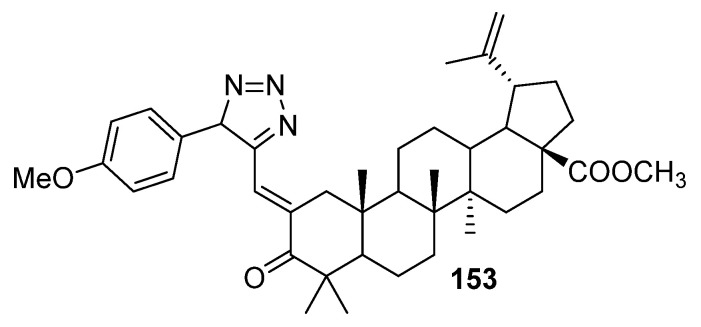
Methyl betulonate C2-substituted triazole.

By a base-promoted aldol condensation of methyl betulonate with 3-(4-methoxyphenyl)propioaldehyde methyl (*E*)-2-[3-(4-methoxyphenyl)-prop-2-yne-1-ylidene]betulonate, **153** was obtained (Figure 48) [62]. Subsequent 1,3-dipolar cycloaddition with NaN_3_ led to the C2-1,2,3 triazole (no data of biological activity were reported).

### 3.5. A-Seco-Triazolyl-Triterpenoids

As one can conclude, the triterpenes involved in the CuAAc reaction mostly have native scaffolds and are functionalized at their side chains. At the same time, triterpenes with a modified A-ring, for example, A-seco derivatives, have rarely been studied.

**Figure 49:**
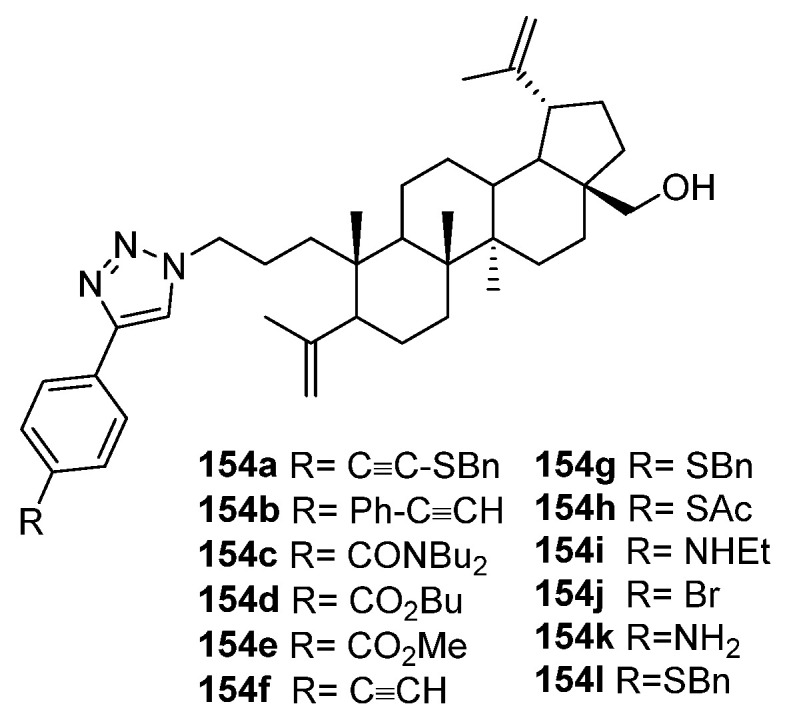
2,3-Seco-dihydrobetulin C3 1,2,3-triazoles.

A-seco-lupane azides were conjugated to phenylalkynes with different substituents in the para position under CuAAC conditions to produce 1,2,3-triazoles **154a–154l** (Figure 49). The compounds were inactive against cancer cell lines and healthy fibroblasts. Triazole **154l** exhibited cytotoxic activity against CEM and MCF-7 cancer cell lines (IC_50_ values of 7.6 µM and 6.5 µM, respectively); however, it was also toxic to normal human BJ fibroblasts (IC_50_ value of 10 µM) [63].

**Figure 50:**
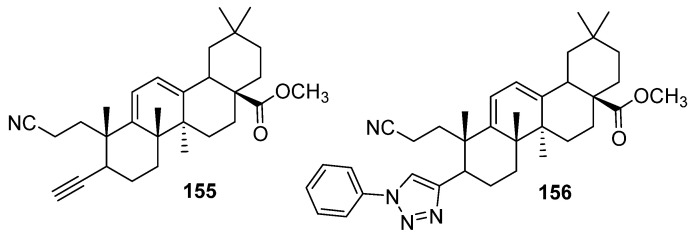
A-Seco-oleanolic acid C4 alkyne and 1,2,3-triazole.

Starting from A-seco-OA C4-alkyne **155** by reaction with azidobenzene under CuAAC reaction conditions, 1,2,3-triazole **156** was obtained (Figure 50) [64].

## 4. Modification of Triterpene Alkynes by the Mannich Reaction

The classic Mannich reaction, a three-component condensation between structurally different substrates containing at least one active hydrogen atom, an aldehyde (mostly paraformaldehyde), and a secondary amine, results in a class of compounds known as Mannich bases [65]. A lot of Mannich bases exhibit biological activity. In addition, aminomethylation of pharmacologically relevant agents can be used to improve their delivery to the human body by increasing their hydrophilic properties through introducing a polar function into their structure. Moreover, aminomethylated compounds can act as prodrugs, releasing the active substance under controlled conditions through hydrolytic deaminomethylation or deamination.

**Scheme 2:**
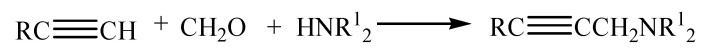
General scheme of the Mannich reaction.

**Scheme 3:**
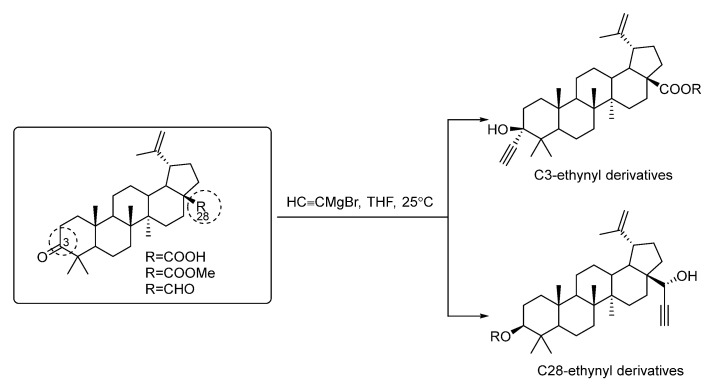
The main method to synthesize C3/C28 ethynyl derivatives.

It should be noted that the Mannich reaction using alkynes as CH acids, aldehydes, and amines leads to the formation of propargylamines (Scheme 2) [66]:

Compared to triazoles, Mannich bases conjugated with triterpene scaffolds are less represented. The main key positions for modification, as in the case of triazoles, are C3 and C28. The first Mannich bases were synthesized from the alkynes obtained by the reaction of the carbonyl carbon atom with organometallic derivatives of acetylene (Scheme 3) [67]. The next examples were obtained from propargylated indoles via the NH group or by nucleophilic substitution reactions at the carbonyl group and amidation via the acyl chloride method (Scheme 1, Figure 3)

**Figure 51:**
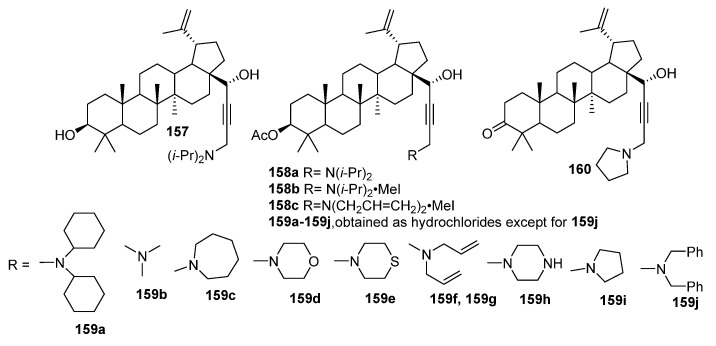
Betulinic acid C28-hydroxypropargylamines.

C28-Ethynyl-triterpenoids were involved in the Mannich reaction with various secondary amines and paraformaldehyde in the presence of CuI and NaOAc [68]. Hydroxypropargylamines (Figure 51) were screened for their antitumor activity in a panel of nine human cancer cell lines in a sulforhodamine B (SRB) assay. Mannich products with aliphatic amines showed the highest cytotoxicity and steric factors played a decisive role. Dicyclohexyl derivative **159a**, as well as dibenzyl compound **159j**, demonstrated a decrease in activity. Moreover, conversion of compounds **158a** and **159f** into salts **158b** and **158c** by interaction with MeI increased the activity by 2.5–5.8 times compared to parent compounds.

**Figure 52:**
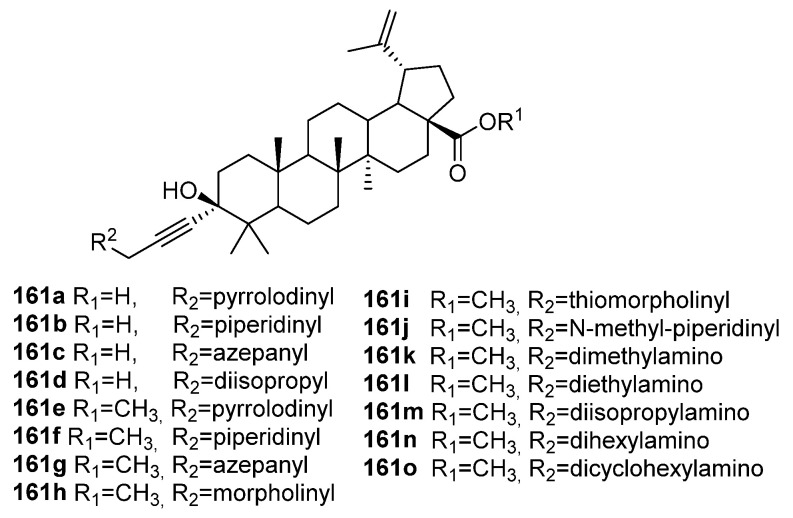
Betulinic acid C3 hydroxypropargylamines.

Lupane-type C3-modified Mannich bases **161a–161o** were obtained and tested for cytotoxicity (Figure 52) [67]. The highest activity was found for the *N*-methyl-piperidinyl derivative **161j**, with IC_50_ values between 2.5 and 5.8 µM for the different human cancer cell lines. Bulky and more hydrophobic substituents (as in compounds **161i** or **161o**) resulted in reduced activity.

**Figure 53:**
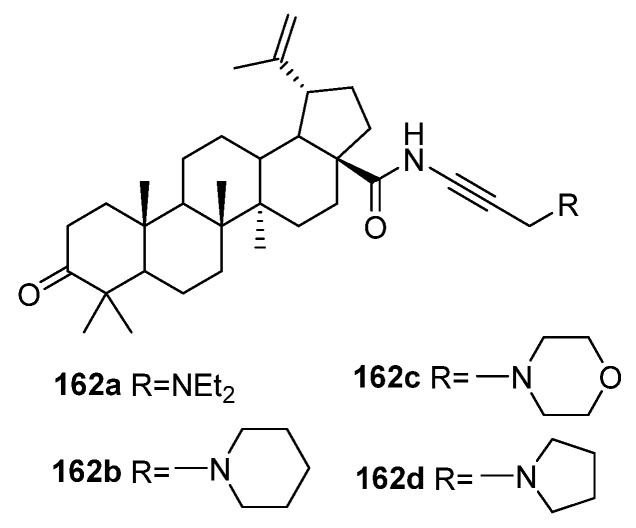
Betulonic acid C28 Mannich bases.

Through a reaction of betulonic acid propargyl amide with secondary amines under Mannich reaction conditions, four propargylamines (**162a–162d**) were synthesized (Figure 53) [69]. No data on biological activity were provided.

**Figure 54:**
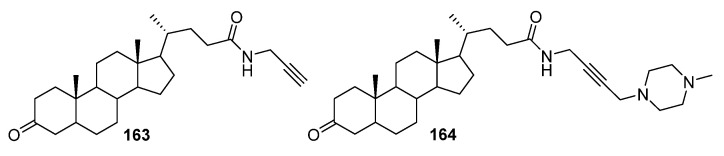
Lithocholic acid alkynyl and propargylaminomethyl derivatives.

This approach was also successfully implemented on lithocholic acid [70]. The conjugate of 3-oxo-lithocholic acid with *N*-methylpiperazine and paraform was synthesized using the Mannich reaction and evaluated for antiviral activity (Figure 54). This modification resulted in a dramatic increase in antiviral activity combined with a two-fold decrease of toxicity. Together, these effects led to a strong increase in the selectivity of the compound (SI value of 40 vs. 3 for compounds **164** and **163**, correspondingly).

**Figure 55:**
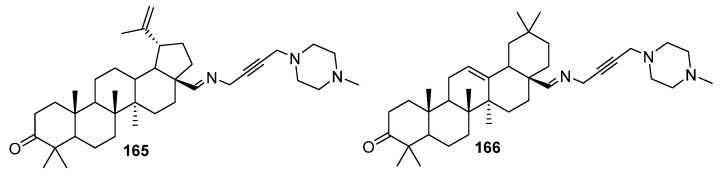
Aminoalkylated *N*-propargyl triterpene aldimines.

The possibility of introducing a propargyl fragment into a triterpene molecule by condensation of propargylamine with triterpene aldehydes and subsequent modification of the terminal alkyne fragment afforded new Mannich bases with an *N*-methylpiperazinyl substituent also present on the lupane and oleanane scaffolds (Figure 55) [10].

**Figure 56:**
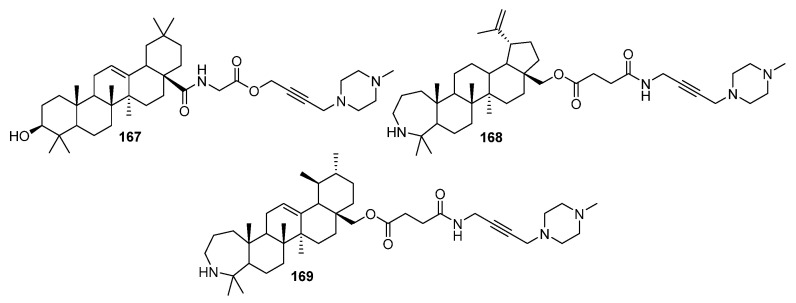
C28-spaced via amino acid or succinic anhydride triterpenic Mannich bases.

There are examples of hybrid C28-modified Mannich bases in which the secondary amine fragment is linked to the triterpenic scaffold through various spacers. Thus, C28 O-propargyl glycinamide of OA conjugated with *N*-methylpiperazine **167** was synthesized (Figure 56). Anticancer activity evaluation showed that **167** suppressed the growth of leukemia (SR) cancer cells [71].

Aminomethylation possibility was demonstrated on succinic acid-tethered azepano-triterpenoids [72]. Stepwise conjugation of biologically active azepanobetulin and azepanouvaol with succinic anhydride and propargylamine, followed by the copper-catalyzed Mannich reaction, afforded new hybrids with *N*-methylpiperazine (**168** and **169**) (Figure 56).

**Figure 57:**
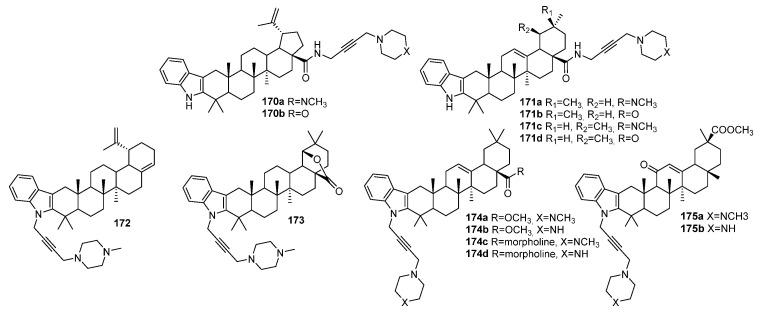
Triterpenic *N*-aminomethylated indoles.

The indole scaffold is a critical and sought-after structural fragment in medicinal chemistry and organic chemistry. Indole-fused molecules are frequently encountered in natural products and demonstrate a wide range of biological activities, such as antibacterial, antitumor, antiplasma, and cholinesterase inhibitory effects [73]. Indolo-triterpenoids have been also introduced for the Mannich reaction using C28 or *N*H indole key positions (Figure 57). Compounds **170–171**, possessing a prorapgyl amide at C28, were aminomethylated with *N*-methylpiperazine or morpholine in the presence of paraform, NaOAc, and CuI [74]. On the other hand, derivatives were obtained from *N*H-propargylated indoles. The Mannich bases **172** and **173** were obtained by successive reactions of indolo-acids with propargyl bromide in the presence of NaH and the Mannich reaction with *N*-methylpiperazine [11]. Later, analogs based on OA and GA (**174** and **175**) were obtained [12]. Among all compounds, **170a**, **171a**, and **171c** with the *N*-methylpiperazine core were active against leukemia, colon cancer, non-small cell lung cancer, and melanoma cells, with the percentage of growth between −25.3% and 29.8%. Compound **171a** was the most selective against the leukemia cell line SR (−2.2%) and non-small cell lung cancer cell line NCI-H460 (−25.3%). The morpholine-containing derivatives **170b**, **171b**, and **171d** were not active, as well as parent propargylamides. *N*-methylpiperazinemethyl-2,3-indolo-oleanolic acid-28-amide **171a** exhibited alpha-glucosidase inhibitory properties, acting as a non-competitive inhibitor, with a K_i_ value of 3.01 µM. Compound **171a** could also alleviate oxidative stress, inflammation, and hyperglycemia—key pathophysiological changes in type 2 diabetes [75]. The Mannich bases of oleanolic and glycyrrhetic acid *N*-propargylated indoles **174a**, **174b**, and **175b** were the most efficacious against influenza virus A, with IC_50_ values of 7–10 μM, together with low toxicity (CC_50_ > 145 μM) and a high selectivity index SI value of 20. The indolo-oleanolic acid morpholine amide Mannich base possessing the *N*-methylpiperazine moiety (**174c**) showed anti-SARS-CoV-2 pseudovirus activity, with and EC_50_ value of 14.8 μM.

**Figure 58:**
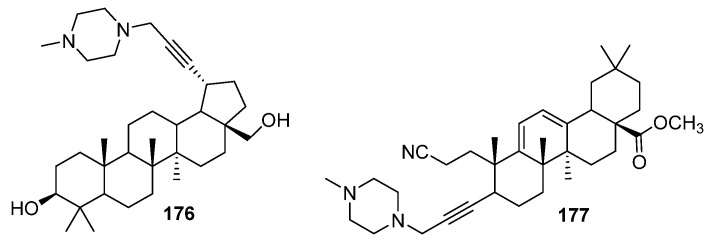
Non-trivial C19 and A-seco Mannich bases.

Non-trivial positions such as C19 of the isopropenyl group or C4 of the A-seco backbone have been also used to synthesize Mannich bases. C19 alkyl-betulin, available from the reaction of messagenin with phosphorus chloride [9], was conjugated with *N*-methylpiperazine to yield compound **176** (Figure 58), which showed manifest anticancer activity against one line of leukemia cells and two lines of colon cancer cell [71].

The synthesis of a Mannich base of methyl 2-cyano-3,4-seco-4(23)-en-olean-28-oate was described [64]. By the reaction of A-seco-C4-alkynyl-containing derivatives of OA with *N*-methylpiperazine by the Mannich reaction (paraformaldehyde, NaOAc, CuI, and 1,4-dioxane), propargylaminoalkyl derivative **177** was obtained (Figure 58).

## 5. Pharmacological Activity

Previous reviews on triazole triterpenoids were mostly focused on their antitumor and antiviral properties [7,8], but recent studies have significantly expanded the range of therapeutic possibilities of this class of compounds. Along with the continuing relevance of antitumor and antiviral activity, increasing attention is being paid to their neuroprotective, antiparasitic, antimicrobial, and antifungal effects (Table 1). In the field of antidiabetic research, there has been a change from the study of glycogen phosphorylase inhibition to alpha-glucosidase inhibition with derivatives possessing triazole introduced via an ester bond being the most potent. For example, the lowest IC_50_ value (0.22 µM) was achieved for the triazole linked to boswellic acid, acting as a competitive inhibitor. On the other hand, triazole attached to C3 of betulinic acid (**103**) showed a mixed type of inhibition [39]. Compound **103** reduced in vivo fasting blood glucose levels in mice, improved glucose tolerance, and reversed dyslipidemia.

Most triterpene derivatives with antitumor activity are characterized by the presence of a triazole moiety at position C28, linked to the triterpene backbone by an amide or ester bond. The emergence of H1N1 antiviral activity is apparently associated with the presence of a bulky cyclodextrin substituent at C28. The influenza virus inhibitory capability is mainly due to action on the hemagglutinin protein through preventing the attachment of the virion to its receptors on host cells and subsequent infection. Among all, the lowest IC_50_ value (2.86 µM) was demonstrated for a complex of glycyrrhetic acid (**50a**), in which cyclodextrin was conjugated to the GA molecule through a triazolo-piperazine spacer. Compound **50a** inhibited H1N1 virus-induced agglutination of red blood cells in a dose-dependent manner at concentrations of 1.3 μM or more. The derivative of ursolic acid (**2**) with a nitro-phenyl substituted in the triazole ring may act similarly to inosine monophosphate dehydrogenase inhibitors and the active metabolite of ribavirin, leading to GTP depletion in the micromolar range (0.053 µM).

The majority of the derivatives obtained over the last ten years have been studied for cytotoxicity against a wide range of cancer cell lines, including breast cancer, lung cancer, glioma, leukemia, and melanoma. The mechanisms of action include activation of the mitochondrial apoptotic pathway, affinity to MDM2 binding sites, inhibition of NF-κB, and induction of necroptosis, cell cycle arrest, and autophagy. However, there is no clear pattern of the structure–activity relationships, since cytotoxic effects were observed in both compounds with simple substituents (for example compounds **13b** and **112j**) in the triazole ring and in hybrid molecules (like **29**, **125b**, and **129d**). At the same time, low values of the inhibitory concentration were observed for compounds with the triazole substituent at the C28 position and by annulation with the A-ring. The examples are derivatives of betulinic (**10a**) and ursolic (**13b**) acids, with IC_50_ values of 1.20 and 1.55 µM for breast cancer, respectively. For compound **10a**, the mechanism of activity was associated with activation of the mitochondrial apoptotic pathway, while for **13b**, the affinity to Mdm2 binding sites was proposed. By contrast, lupane-type derivatives modified at the C30 position were weakly active.

In the case of antiparasitic activity, the **GA** derivative **30n** with a triazole ring at C30 exhibited an inhibitory effect against the chloroquine-sensitive (NF-54) strain of *P. falciparum*, with an IC_50_ value of 0.47 µM (in vivo), by decreasing the metabolic activity in *P. falciparum* in a dose-dependent manner [23].

**Figure 59:**
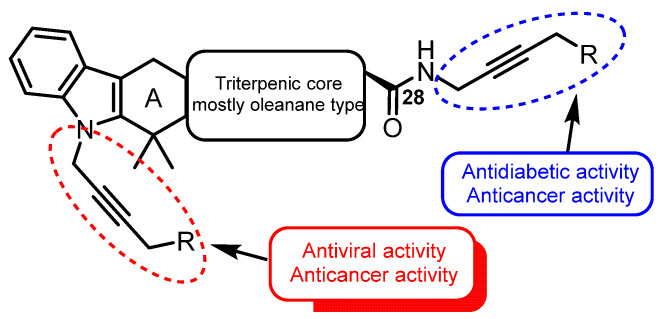
The main directions of the activity of triterpene Mannich bases.

All of the obtained Mannich bases, as well as triazolyl derivatives, were mainly studied for antitumor activity, with the exception of some indole derivatives, for which antiviral and antidiabetic activity was found. It was shown that aminomethylation of the alkynyl group on the indole ring has a positive effect on antiviral properties, while C28 analogs have greater antidiabetic potential (Figure 59). The emergence of antitumor properties is characteristic of both NH and C28 modifiers. In addition, the key factor is the type of amine; for example, only *N*-methyl-piperazine and piperazine had positive effects, while morpholine and *N*-ethyl-piperazine did not. For example, the most active was 2,3-indolo-oleanolic *N*-methyl-piperazinemethyl-28-amide (**171a**), which exhibited α-glucosidase inhibitory properties acting as a non-competitive inhibitor, with a Ki value of 3.01 µM, as well as the abilities to alleviate oxidative stress, inflammation, and hyperglycemia—key pathophysiological changes in type 2 diabetes. Among indoles propargylated at the NH group, oleanolic and glycyrrhetinic acid derivatives **174a**, **174b**, and **175b** were the most effective against influenza A virus, with IC_50_ values of 7 and 10 µM at low toxicity (CC_50_ > 145 µM), respectively. The study of antitumor activity revealed a derivative (**174c**) that selectively acted on the cell lines of leukemia K-562 (−23.00%) and melanoma LOX IMVI (−46.15%). In addition, compound **175a** was active against colon cancer SW-620 (−63.43%) and melanoma LOX IMVI (−91.62%).

Taking into account the above discussion, we can conclude that the specific modifications of the triterpene core at the A-ring or free carboxyl group regulate the type and spectrum of biological activity, which can help to develop new molecules with desired properties.

## 6. Conclusions and Perspectives

In this review, we have analyzed the progress in the synthesis of 1,2,3-triazole derivatives and Mannich bases conjugated to a triterpene scaffold over the period of 2019 to 2024. Despite the synthesis of a significant number of derivatives, the main focus was on the study of their antitumor and antiviral activities, while their antibacterial, anti-inflammatory, and antidiabetic properties were studied to a lesser extent. Acylation of hydroxyl and carboxyl groups using propargyl bromide or propargylamine in the presence of base catalysis remains the main method to introduce a triple bond into the molecule. However, over the past five years, modifications of ring-fused triazoles or C28 (or C30)-substituted triterpenoids have continued, expanding the library of triazoles. For the first time, the possibility of *N*-alkylation of the indole ring of triterpenoids by propargyl bromide to obtain *N*-substituted derivatives was demonstrated. The reaction of the aldehyde group at C28 with propargylamine has been proposed as a new method for the synthesis of triterpene aldimines, which are rare cases in triterpene modification. In addition, this review is the first to collect information including modification and biological data of alkynyl triterpenoids using the Mannich reaction method. Aminomethylation involved functionalization at the C28 carboxyl group, the indole amino group, the C19 position of betulin, and the introduction of a secondary amine through amino acid spacers.

The results presented in the current review clearly show that the application of various methods makes it possible to adjust the activity. For example, complexation with cyclodextrins can significantly increase the antiviral activity of triterpenoids, while the introduction of smaller substitutes affects the occurrence of antidiabetic activity. More attention has been paid to hybrid molecules that contain long-chain substituents, such as PROTAC conjugates with cytotoxic activity. The idea to synthesize ribbons led to derivatives with antimicrobial activity, as well as derivatives with a spermine-polyamine fragment. Future research should be focus on elucidating the detailed molecular mechanisms of biological activity as well as exploring the potential in preclinical and clinical settings.

## Figures and Tables

**Table 1:** The structure and biological activity data of the most active 1,2,3-triazole-derivatives.

No.	Parametrs of Activity and Mechanism of Action	Ref.
Antidiabetic activity		
**22e**	PTP1B enzyme inhibitory activity IC_50_ 4.15 μM	[19]
**34f**	A-glucosidase inhibitory activity IC_50_ 0.22 µM; K_i_ 0.84 µM Exhibited a competitive type of inhibition: the inhibitor binds with the active site residue of the enzyme. The molecular docking interactions indicated that all compounds were well fitted in the active site of α-glucosidase, where His280, Gln279, Asp215, His351, Arg442, and Arg315 mainly stabilize the binding of these compounds.	[25]
**103**	A-glucosidase inhibitory activity IC_50_ 2.83 ± 0.19 μM A mixed type inhibitor, interacts with α-glucosidase by changing the secondary structure of the protein, could bind to the active site of α-glucosidase (molecular docking studies), could reduce fasting blood glucose levels in mice and improve glucose tolerance and reverse dyslipidemia (in vivo).	[39]
Neuroprotective activity		
**142**	Modulated oxidative stress and caspase-3,7 activity, blocked mitochondrial permeability transition pore opening, demonstrated potent restoration of the mitochondrial membrane potential.	[52]
Antiviral activity		
**2**	Anti-RSV activity EC_50_ 0.053 µM May act similarly to inosine monophosphate dehydrogenase inhibitors and the active metabolite of ribavirin, leading to guanosine triphosphate depletion.	[13]
**36a**	Anti-influenza activity against A/WSN/33 (H1N1) IC_50_ 5.20 µM Inhibited viral replication by directly targeting the influenza hemagglutinin protein (KD 1.50 μM), thus efficiently preventing the attachment of the virion to its receptors on host cells and subsequent infection.	[26,27]
**43, 45**	Anti-influenza activity against A/WSN/33 (H1N1) 100% inhibition	[28]
**50a**	Anti-influenza activity against A/WSN/33 (H1N1) IC_50_ 2.86 μM Inhibited influenza A/WSN/33 (H1N1) virus-induced agglutination of cRBCs (red blood cells) in a dose-dependent manner at concentrations of 1.3 μM or more. Similar results were observed for the anti-hemagglutinin antibody at concentrations of 0.063 ng/mL or more.	[29]
**65a**	Anti-influenza activity against A/WSN/33 (H1N1) IC_50_ 5.47 µM Hemagglutination inhibition assay and docking simulation indicated that the antiviral activity was potentially due to its high affinity to HA protein, thus blocking the attachment of influenza viruses to host cells.	[33]
**67a**	Anti-influenza activity against A/WSN/33 (H1N1) IC_50_ 5.23 μM Could disrupt the interaction between protein hemagglutinin and its sialic acid receptor, thus blocking virus and host cell recognition.	[34]
**128e**	Anti-HIV-1 EC_50_ 50.6 µM	[50]
**148a, 148c**	Antiviral activity against human papillomavirus-11 R = H: EC_50_ < 2.97 µM, SI > 125 R = F: EC_50_ 1.20 µM, SI > 7	[60]
**150b**	Antiviral activity against human cytomegalovirus EC_50_ < 0.05 µM, SI 81	[60]
Antiparasitic, antimicrobial, and antifungal activities		
**6b**	Growth-stimulating activity against strains in the range of 20–200%	[15]
**25d**	Antiparasitic activity against *T. gondii* IC_50_ 128.0 µM Possessed a strong binding affinity for *T. gondii* calcium-dependent protein kinase 1.	[20]
**30n**	Antiplasmodial activity against chloroquine-sensitive (NF-54) strain *P. falciparum.* IC_50_ 0.47 µM (in vivo) Increased oxidative stress and decreased the metabolic activity of *P. falciparum* in a dose-dependent manner, induced apoptosis-like cell death (the loss of mitochondrial membrane potential, activation of caspase proteases, and DNA damage).	[23]
**79c**	Antifungal activity against *S. sclerotiorum* 89.6% inhibition	[39]
**111g**	Antibacterial activity against *L. monocytogenes* MIC_50_ 9.48 µM The in silico molecular docking study revealed that the compound can fit well into the binding cavity of the ABC substrate-binding protein Lmo0181 from *L. monocytogenes*.	[41]
**120c**	Antibacterial activity against *E. faecalis*, *E. coli*, and *P. aeruginosa* MIC 3.25 µg/mL (for all strains)	[45]
**124a**	Antimicrobial activity against *L. acidophilus* IC_50_ < 1.13 µM Proposed mechanism of action is based on the disturbing of the membrane integrity of bacteria by interaction with negative charges of phosphate groups of the LPS on the surface of the outer membrane.	[47,48]
**128e**	Antimicrobial inhibitory activity against *S. aureus* and *E. coli* >50% (c 25 µg/mL)	[50]
**134f**	Antimicrobial inhibitory activity against *S. aureus* >40% (c 25 µg/mL)	[50]
**130**	Antimicrobial inhibitory activity against *E. faecalis* >62% (c 25 µg/mL)	[50]
**135c**	Antimicrobial activity against *S. enterica subsp. enterica * MIC_50_ 16.61 mg/mL	[51]
Anti-inflammatory activity		
**22b**	Anti-inflammatory activity COX-2 inhibition IC_50_ 1.16 µM, SI 64.66	[21]
**33b**	Suppressed the expression of pro-inflammatory cytokines including IL-6, TNF-α and NO, it also suppressed the expression of iNOS and COX-2 in LPS-induced RAW264.7 cells in a dose-dependent manner. Western blot results indicated that the suppressing effect on pro-inflammatory cytokines was correlated with the suppression of NF-κB and MAPK signaling pathways.	[24]
Anticancer activity		
**13b**	Cytotoxicity against breast cancer cell line MCF-7 IC_50_ 1.55 µM Molecular docking data supported by flow cytometry analysis showed that the most likely mechanism of cytotoxic activity was the affinity to Mdm2 binding sites.	[18]
**10a**	Cytotoxicity against breast cancer cell line T47D IC_50_ 1.20 µM Activated the mitochondrial apoptosis pathway in A549 cells. The molecular docking study showed that the interaction of the 1,4-quinone moiety with the enzyme active site through hydrogen bond led to an increase in activity.	[16]
**29**	Anticancer activity against A549 (IC_50_ 1.89 µM), Huh7 (IC_50_ 1.87 µM), and HepG2 (IC_50_ 1.77 µM) cell lines. Induced significant degradation of MDM2 (only 25% to that of SM1) and promoted the expression of P21 and PUMA proteins, thus inhibiting the proliferation (77.67% of 1B vs. 60.37% of CON in G1 phase) and promoting the apoptosis (26.74% of 1B vs. 3.35% of CON) of A549 cells.	[22]
**60c**	Cytotoxicity against all cancer cell lines IC_50_ range 0.60–1.60 µM Apoptosis via the mitochondrial pathway, inhibited growth and disintegrated spheroid cultures of HCT116 and HeLa cells, which would be important for the treatment of solid tumors.	[31]
**68, 69**	Antiproliferative activity against ovarian cancer cell line A2780 IC_50_ 5.9 µM for **68**; IC_50_ 9.4 µM for **69** Could inhibit cell metastasis and invasion through damage of actin dynamics and downregulation of MMP2/MMP9 proteins. Mitochondrial accumulation of metal-arene complexes caused mitochondrial membrane potential damage, oxidative phosphorylation, ATP depletion, and autophagy. Displayed excellent activity to disintegrate 3D multicellular tumor spheroids, showing potential for the treatment of solid tumors.	[35]
**71k**	Cytotoxicity against lung cancer cell line A549 IC_50_ 2.67 µM Inhibited NF-κB DNA binding, the activity of NF-κB, nuclear translocation, and IκBα phosphorylation. The treatment of A549 cells induced apoptosis and inhibited in vitro cell migration. May be a potential NF-κB inhibitor with the ability to induce apoptosis and suppress cell migration.	[36]
**73i**	Cytotoxicity against glioma cell line U251 IC_50_ 0.94 µM Induced necroptosis mainly by activating the RIP1/RIP3/MLKL pathway, exerted acceptable BBB permeability in mice, and inhibited U251 cell proliferation in an in vivo zebrafish xenograft model.	[37]
**112j**	Cytotoxicity against breast cancer cell line MDA-MB-231 IC_50_ 4.45 µM Can target CHK1 to produce anticancer effects in TNBC (triple negative breast cancer).	[42]
**113a**	Cytotoxicity against lung cancer cell line A549 IC_50_ 7.19 µM Induced G1 phase arrest in A549 cell lines, regulated the signal transducer and activator of transcription 3 signal pathway by inhibiting phosphorylation of JanusKinase 2 and STAT3 and simultaneously increasing the protein level of downstream cyclin p21.	[43]
**123a**	Cytotoxicity against human T-lymphoblastic leukemia cancer cell line IC_50_ 6.5 µM	[46]
**125b**	Cytotoxicity against breast cancer cell line MCF-7 IC_50_ 3.0 µM	[49]
**129d**	Cytotoxicity against melanoma cancer cell line G-361 IC_50_ 20.0 µM	[50]
**136f**	Cytotoxicity against leukemia cell line HL-60 cells IC_50_ 1.3 µM Caused mitochondrial dysfunction and arrested the cell cycle in the G0/G1 phase to induce apoptosis of HL-60 cells, also induced autophagy and inhibited the proliferation of HL-60 cells.	[51]
**141**	Cytotoxicity against Hela (IC_50_ 1.19 µM), Hep92 (IC_50_ 1.15 µM), MCF-7 (IC_50_ 1.14 µM), MDA-MB-231 (IC_50_ 1.12 µM), and SGC-7901 (IC_50_ 1.05 µM) cells Could induce SGC-7901 cell apoptosis through the mitochondrial pathway, inducing cell cycle arrest. Could reduce the expression of Vimentin and increase the expression of E-cadherin to hinder the epithelial–esenchymal transition.	[52]
**144a**	Cytotoxicity against melanoma cancer cell line RPMI 88.3% inhibition at a dose of 2 µM May induce apoptosis by disrupting the Bcl2/Bax ratio.	[54]
**144b**	Cytotoxicity against melanoma cancer cell line RPMI-7951 IC_50_ 18.8 µM Revealed a mitochondria-level induced apoptotic mechanism, inhibited mitochondrial respiration in RPMI−7951 cells, caused a significant decrease of anti-apoptotic Bcl-2 gene expression, while increasing pro-apoptotic BAX gene expression.	[55]
**145a**	Cytotoxicity against breast cancer cell line MCF-7 IC_50_ 4.37 µM	[56]
**152d**	Antiproliferative activity against human cancer cell lines SMMC-7721 (IC_50_ 5.57 µM), HepG2 (IC_50_ 7.49 µM), MNK-45 (IC_50_ 6.31 µM), SW620 (IC_50_ 6.00 µM), and A549 (IC_50_ 5.79 µM) Induced cell apoptosis and autophagy in SMMC cells, resulting in antiproliferation and G0/G1 cell cycle arrest by regulating protein expression levels of Bax, Bcl-2, and LC3.	[61]
**154l**	Cytotoxicity against breast cancer cell line MCF-7 IC_50_ 6.5 µM	[63]

## Data Availability

The data presented in this study are available upon request from the corresponding author.

## Abbreviations

The following abbreviations are used in this manuscript:

AA	Asiatic acid
AZT	Azidothymidine
BA	Betulinic acid
BE	Betulin
CC50	Cytotoxicity concentration
CuAAC	Copper-catalyzed azide-alkyne cycloaddition
DCC	*N*,*N*′-Dicyclohexylcarbodiimide
DIPEA	*N*,*N*-Diisopropylethylamine
DIEA	*N*,*N*-Diisopropylethylamine
DMAP	4-Dimethylaminopyridine
DMF	Dimethylformamide
EDCI	1-Ethyl-3-(3-dimethylaminopropyl)carbodiimide
GA	Glycyrrhetinic acid
GTP	Guanosine triphosphate
HATU	Hexafluorophosphate azabenzotriazole tetramethyl uronium
HCMV	Human cytomegalovirus
HOBt	Hydroxybenzotriazole
HPV-11	Human papillomavirus 11
HSV-1	Herpes simplex virus 1
IC_50_	Half maximal inhibitory concentration
MA	Maslinic acid
MoA	Morolic acid
NBS	*N*-Bromosuccinimide
OA	Oleanolic acid
POE	Polyoxyethylene
PTP1B	Protein-tyrosine phosphatase 1B
RSV	respiratory syncytial virus
rtPCR	reverse transcription polymerase chain reaction
TEA	Tetraethylammonium
UA	Ursolic acid
β-CD	β-Cyclodextrin
β-KBA	β-11-keto-boswellic acid
